# 56-year trends and disparities in systemic sclerosis mortality in the United States, 1968–2023: a retrospective population-based study

**DOI:** 10.1093/rap/rkag107

**Published:** 2026-08-31

**Authors:** Yanhua Xiao, Zhaolin Zeng, Jie Liu, Zhifei Deng, Rui Chen, Dibin Guo, Xuezhi Hong

**Affiliations:** Department of Dermatology, The First Affiliated Hospital of Gannan Medical University, Ganzhou, China; Department of Dermatology, The First Affiliated Hospital of Gannan Medical University, Ganzhou, China; Department of Dermatology, The First Affiliated Hospital of Gannan Medical University, Ganzhou, China; Department of Dermatology, The First Affiliated Hospital of Gannan Medical University, Ganzhou, China; First Clinical Medical College, Gannan Medical University, Ganzhou, China; First Clinical Medical College, Gannan Medical University, Ganzhou, China; Department of Rheumatology and Immunology, The First Affiliated Hospital of Gannan Medical University, Ganzhou, China; Department of Rheumatology and Immunology, The First Affiliated Hospital of Gannan Medical University, Ganzhou, China

**Keywords:** systemic sclerosis, underlying cause of death, age adjusted mortality rates, annual percent change, average annual percent change, multiple cause of death

## Abstract

**Objectives:**

To describe long-term US trends in SSc mortality and disparities by sex, race, region and age.

**Methods:**

This was a retrospective population-based mortality study using CDC WONDER data from 1968 to 2023. We identified deaths with SSc as the underlying cause of death (UCD) and calculated annual age-adjusted mortality rates (AAMRs) per 1 000 000 population standardized to the 2000 US standard population. Joinpoint regression was used to estimate annual percent change (APC) and average annual percent change (AAPC). Sensitivity analyses used multiple cause of death (MCOD) data (1999–2023).

**Results:**

Among 55 567 SSc-UCD deaths, the annual number increased from 466 in 1968 to 1005 in 2023. The national AAMR increased from 2.66 (1968) to 4.87 (2001) and decreased to 2.37 (2023). Joinpoints occurred in 1987, 2001 and 2013; the steepest decline followed 2013 (APC −4.07%), with an overall AAPC of −0.44%. Female AAMR exceeded male AAMR throughout (female:male ratio from 2.0 to 3.6) and mortality declined more in males than females (AAPC −1.08% *vs* − 0.02%). Black/African American individuals had higher AAMRs than White individuals but a larger net decrease (AAPC −0.80% *vs* − 0.33%). Mortality increasingly concentrated in adults ≥65 years of age, who showed a net increase over 1968–2023 (AAPC 1.07%). Across US census regions, mortality peaked in the early 2000s and declined thereafter, with broadly similar post-peak trajectories. In MCOD sensitivity analyses, AAMRs declined overall from 7.06 to 3.79 during 1999–2023 (AAPC −2.45%), although the most recent MCOD segment did not show a statistically significant decline.

**Conclusion:**

Despite the observed long-term decline in SSc-UCD mortality, deaths remained unevenly distributed by sex, race and age. These findings support risk-stratified follow-up and closer monitoring of older adults, women, Black/African American patients and other patients at elevated risk.

Key messages

- SSc underlying-cause mortality peaked in 2001 and declined steeply after 2013.
- Adults ≥65 years of age are the only demographic showing a net mortality increase.
- Significant mortality disparities persist across sex, race and geographic regions in the USA.

## Introduction

SSc is a multisystem autoimmune connective tissue disease characterized by vascular injury, immune dysregulation and progressive fibrosis of the skin and internal organs [1]. Despite advances in care, SSc remains associated with substantial premature mortality. Death is most often linked to cardiopulmonary involvement, particularly interstitial lung disease (ILD) and pulmonary vascular disease, although gastrointestinal complications, renal crisis and cardiac manifestations also contribute [2–4].

Population-level mortality surveillance remains valuable in SSc. Referral-based cohorts, although clinically informative, may overrepresent severe disease, and long-term mortality trends may also be shaped by changes in diagnostic recognition, access to subspecialty care, coding practice and death certificate completion [5–7]. Previous US death certificate studies provide useful context. A nationwide analysis covering 1968–2015 showed that SSc mortality coded as the underlying cause of death (UCD) increased through 2000 and then decreased, with clear differences by sex and race [8]. Another study limited to the International Classification of Diseases, 10th Revision (ICD-10) era (1999–2017) also found a decrease in age-adjusted mortality and highlighted geographic variation across states [9]. Taken together, these studies suggest that SSc mortality has decreased in the modern era, but that the burden remains unevenly distributed.

The most recent years have not yet been examined within a consistent national framework and extending the analysis beyond 2015 or 2017 is relevant because cardiopulmonary screening practices, referral pathways and treatment strategies have continued to evolve [7, 10]. In addition, it remains unclear whether long-term mortality trajectories differ by age group and US census region across the full observation window. Examining these patterns may help explain how the mortality burden has shifted over time and whether differences in healthcare access or diagnostic intensity are reflected in the observed trends. Because death certificate–based studies are also affected by cause-of-death attribution, analyses based on multiple cause of death (MCOD) data may help place UCD-based trends in context and provide a broader view of the mortality burden linked to SSc [6].

The primary objective of this study was to evaluate the long-term temporal trends and age-adjusted mortality rates (AAMRs) among the US population where SSc was recorded as the underlying cause of death through 2023. Specifically, we aimed to determine whether these mortality trajectories differed significantly across key demographic subgroups (sex, race and age) and US census regions. A secondary objective was to assess the robustness of these trends using MCOD data to account for potential shifts in death certification practices over time.

## Methods

### Study design and setting

This retrospective population-based mortality study used publicly available aggregate mortality data derived from US death certificates. We examined long-term mortality trends in SSc in the USA from 1968 to 2023.

### Data source

We obtained national mortality data from the Centers for Disease Control and Prevention’s Wide Ranging Online Data for Epidemiologic Research (CDC WONDER) for US residents covering 1968–2023. These files are derived from death certificates for the 50 states and the District of Columbia. Beginning in 1999, the data include a multiple-cause format that captures the UCD together with contributing conditions recorded on the certificate. No record linkage was performed and the authors had access only to publicly available aggregate mortality data.

### Participants and eligibility

The primary analysis included all US resident deaths recorded in CDC WONDER from 1968 to 2023 in which SSc was coded as the UCD. For race-stratified analyses, we restricted modelling to Black/African American and White individuals because annual counts in other racial groups were too small to produce stable rate estimates. Because the analyses were based on publicly available aggregate outputs from CDC WONDER, individual-level records were not available and record-level missing data handling was not applicable.

### Case definitions and analytic variables

In the primary analysis, we identified deaths in which SSc was recorded as the UCD, defined as the condition initiating the sequence of events leading directly to death. SSc was identified using ICD codes corresponding to each coding era: ICD-8 code 734.0 (1968–1978), ICD-9 code 710.1 (1979–1998) and ICD-10 code M34 (1999–2023). We summarized SSc-UCD mortality overall and by sex, age at death (≤44, 45–64, ≥65 years) and US census region (Northeast, Midwest, South, West). For sensitivity analyses, we used MCOD data from 1999 to 2023 and defined SSc-MCOD deaths as deaths with any mention of SSc on the death certificate, including SSc recorded as the UCD or as a contributing condition.

### Statistical analysis

Annual AAMRs were calculated by direct standardization to the 2000 US standard population and are reported per 1 000 000 persons. Annual female:male rate ratios were calculated as the female AAMR divided by the male AAMR.

Temporal trends in annual AAMRs were analysed using the Joinpoint Regression Program version 5.4.0. We fitted log-linear models with calendar year as the independent variable and used the annual standard errors exported from CDC WONDER to account for heteroscedasticity; errors were assumed to be uncorrelated. Candidate models were fitted using grid search and the final number and location of joinpoints were selected using the weighted Bayesian information criterion (BIC). Models allowed zero to seven joinpoints for UCD analyses from 1968 to 2023 and zero to four joinpoints for MCOD sensitivity analyses from 1999 to 2023. We required at least two observations before the first joinpoint, after the last joinpoint and between adjacent joinpoints.

Annual percent change (APC) and average annual percent change (AAPC) with 95% CIs were estimated in Joinpoint. The AAPC represents a weighted average of the segment-specific APCs over the specified interval and was calculated from the final selected model. APC and AAPC estimates were considered statistically significant when their 95% CIs excluded zero. MCOD sensitivity analyses were performed overall and by sex to assess the consistency of the overall trend under a broader any-mention definition.

### Ethics, data access and reporting

This study was exempt from ethical approval under the US Common Rule due to the use of de-identified, publicly available aggregate data from CDC WONDER. All analyses were conducted in accordance with CDC WONDER terms of use. The study was not prospectively registered and no formal preregistered protocol was developed.

## Results

### Characteristics of the study population

A total of 55 567 deaths with SSc recorded as the UCD were identified during 1968–2023. Most decedents were female [43 751 (78.74%)]. Deaths were concentrated among older adults, with 28 438 deaths (51.18%) occurring in persons ≥65 years of age. By US census region, the largest number of deaths occurred in the South [20 044 (36.07%)], followed by the Midwest [13 015 (23.42%)], Northeast [11 686 (21.03%)] and West [10 822 (19.48%)].

### Overall trends for SSc

**Figure 1:**
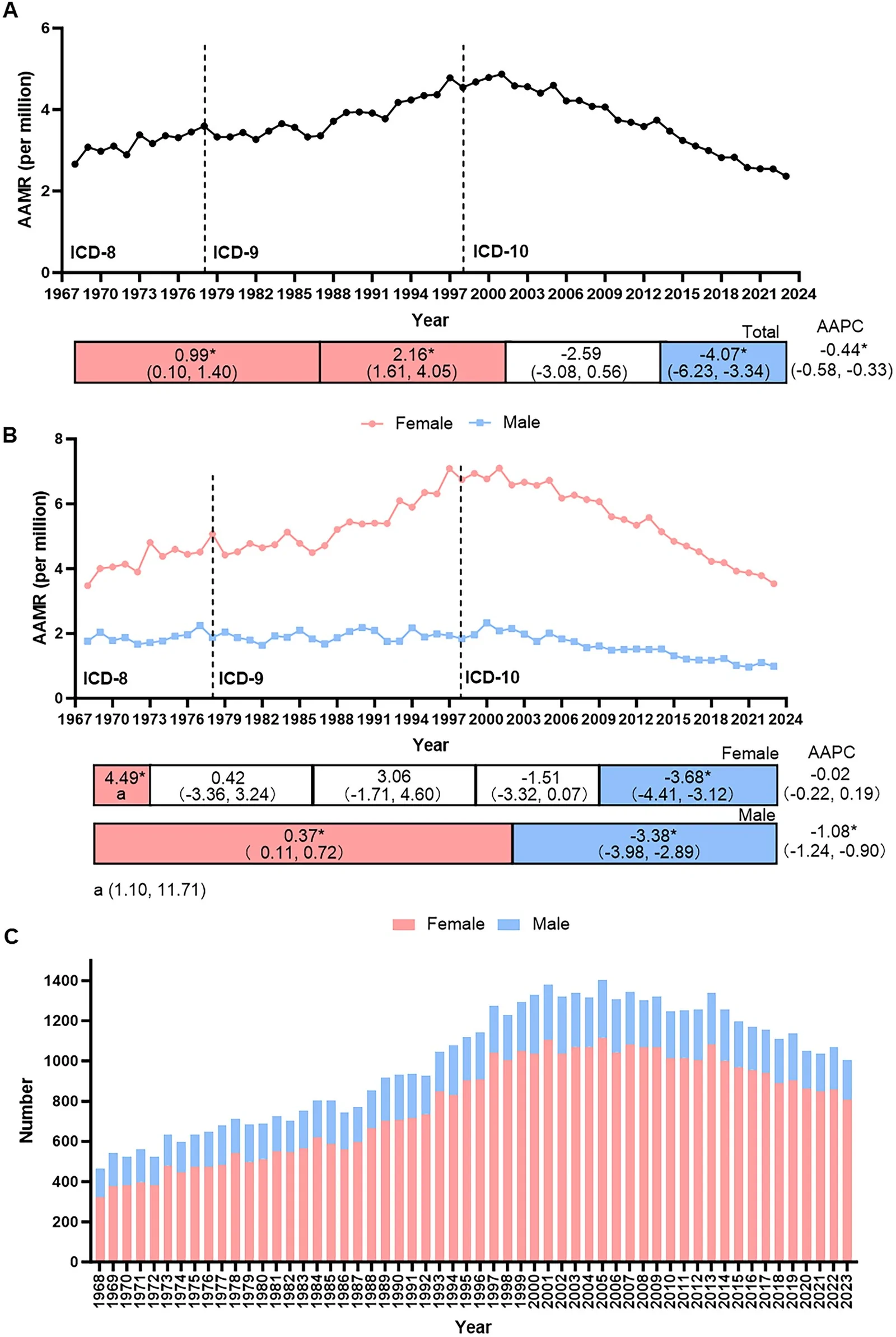
Overall and sex-specific SSc-UCD AAMRs and annual deaths, 1968–2023. **(A)** Annual AAMR (deaths per 1 000 000 persons; standardized to the 2000 US standard population). The coloured line segments beneath the curve depict the Joinpoint-estimated APC with 95% CIs. *Indicates statistical significance. **(B)** Sex-specific AAMR (female and male) with APC (95% CI) displayed in the corresponding segment bars. **(C)** Annual number of SSc-UCD deaths by sex

Overall, SSc-UCD AAMR increased for several decades, peaked in the early 2000s and then decreased, with a faster decrease in the most recent decade. Deaths with SSc as the UCD increased from 466 in 1968 to 1005 in 2023 and peaked in 2005 (1401 deaths). The AAMR increased from 2.66 per 1 000 000 in 1968 to 4.87 in 2001 and then decreased to 2.37 in 2023 (Fig. 1; Supplementary Table S1). Joinpoint regression identified turning points in 1987, 2001 and 2013. Rates increased in 1968–1987 [APC 0.99% (95% CI 0.10, 1.40)] and 1987–2001 [APC 2.16% (95% CI 1.61, 4.05)]. From 2001 to 2013, the estimated slope was negative but not statistically different from zero [APC −2.59% (95% CI −3.08, 0.56)], followed by a significant decrease in 2013–2023 [APC −4.07% (95% CI −6.23, −3.34)]. Over 1968–2023, the overall trend was downward [AAPC −0.44% (95% CI −0.58, −0.33)].

### Trends by sex

Female mortality exceeded male mortality throughout and the gap widened over time. Female AAMRs were higher than male AAMRs in every year and the female:male ratio increased from 2.0 in 1968 to 3.6 in 2023 (Fig. 1; Supplementary Table S1). Female AAMRs peaked at 7.10 in 2001 and decreased to 3.53 in 2023, whereas male AAMRs peaked at 2.33 in 2000 and decreased to 0.98 in 2023. In joinpoint models, females increased in 1968–1973 [APC 4.49% (95% CI 1.10, 11.71)] and decreased after 2009 [APC −3.68% (95% CI −4.41, −3.12)], resulting in little net change overall [AAPC −0.02% (95% CI −0.22, 0.19)]. Males increased before 2002 [APC 0.37% (95% CI 0.11, 0.72)] and decreased thereafter [APC −3.38% (95% CI −3.98, −2.89)], yielding an overall decrease [AAPC −1.08% (95% CI −1.24, −0.90)].

### Trends by race

**Figure 2:**
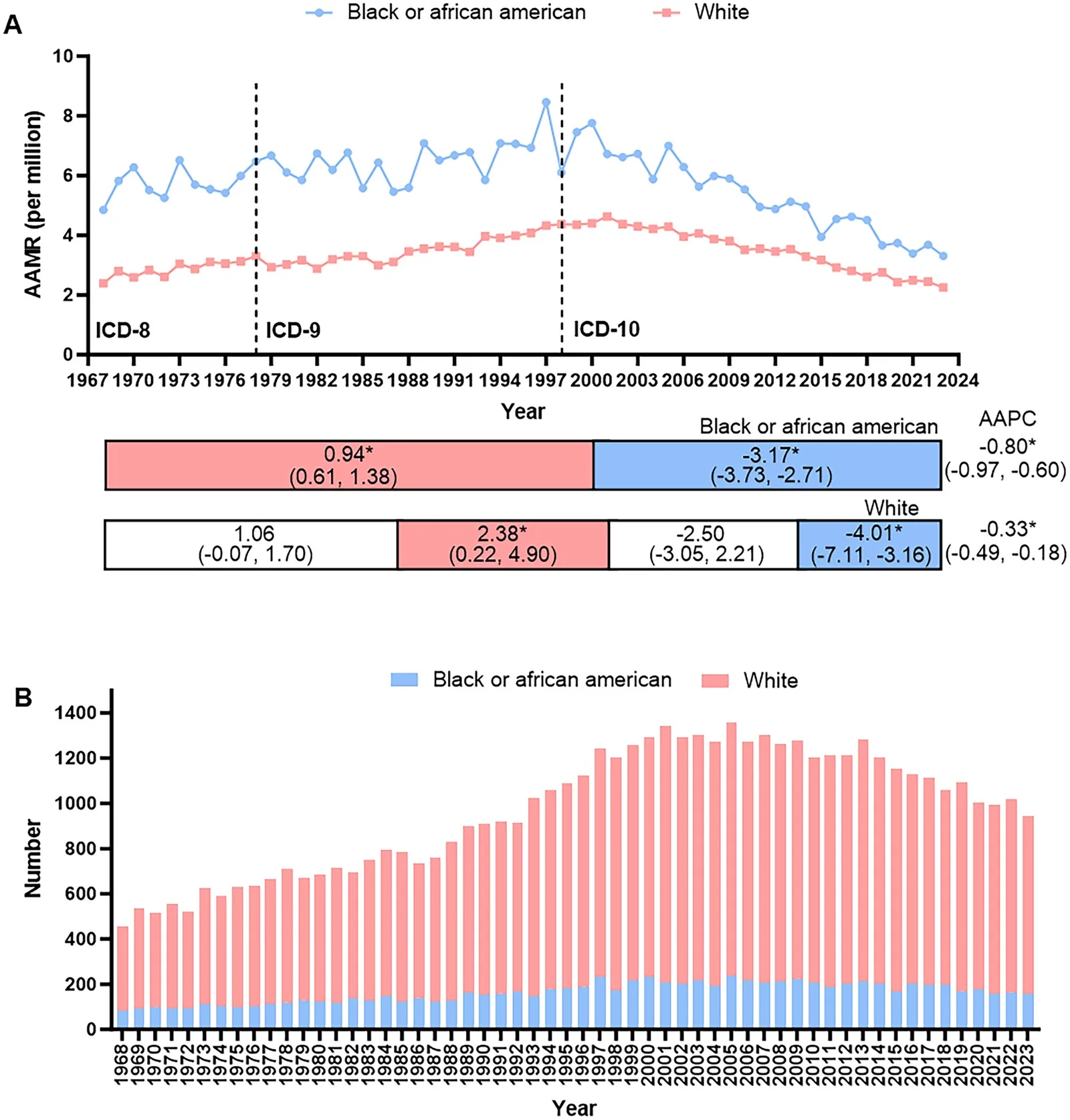
Racial differences in SSc-UCD AAMR, 1968–2023. **(A)** Annual AAMR (per 1 000 000; standardized to the 2000 US standard population) by race. Vertical dashed lines indicate ICD coding eras. Segment bars show APC with 95% CI. *Indicates statistical significance. **(B)** Annual number of SSc-UCD deaths, stacked by race (Black/African American, White)

AAMRs were consistently higher in Black/African American than in White individuals, although the long-term decrease was larger in Black/African American individuals (AAPC −0.80% *vs* − 0.33%; Fig. 2; Supplementary Table S2). In Black/African American individuals, a single turning point occurred in 2000. Rates increased in 1968–2000 [APC 0.94% (95% CI 0.61, 1.38)] and decreased in 2000–2023 [APC −3.17% (95% CI −3.73, −2.71)], with an overall decrease [AAPC −0.80% (95% CI −0.97, −0.60)]. In White individuals, turning points were identified in 1987, 2001 and 2013. Rates changed little in 1968–1987 [APC 1.06% (95% CI −0.07, 1.70)], increased in 1987–2001 [APC 2.38% (95% CI 0.22, 4.90)], did not change significantly in 2001–2013 [APC −2.50% (95% CI −3.05, 2.21)] and decreased in 2013–2023 [APC −4.01% (95% CI −7.11, −3.16)], corresponding to a smaller overall decrease [AAPC −0.33% (95% CI −0.49, −0.18)].

### Trends by US census region

**Figure 3:**
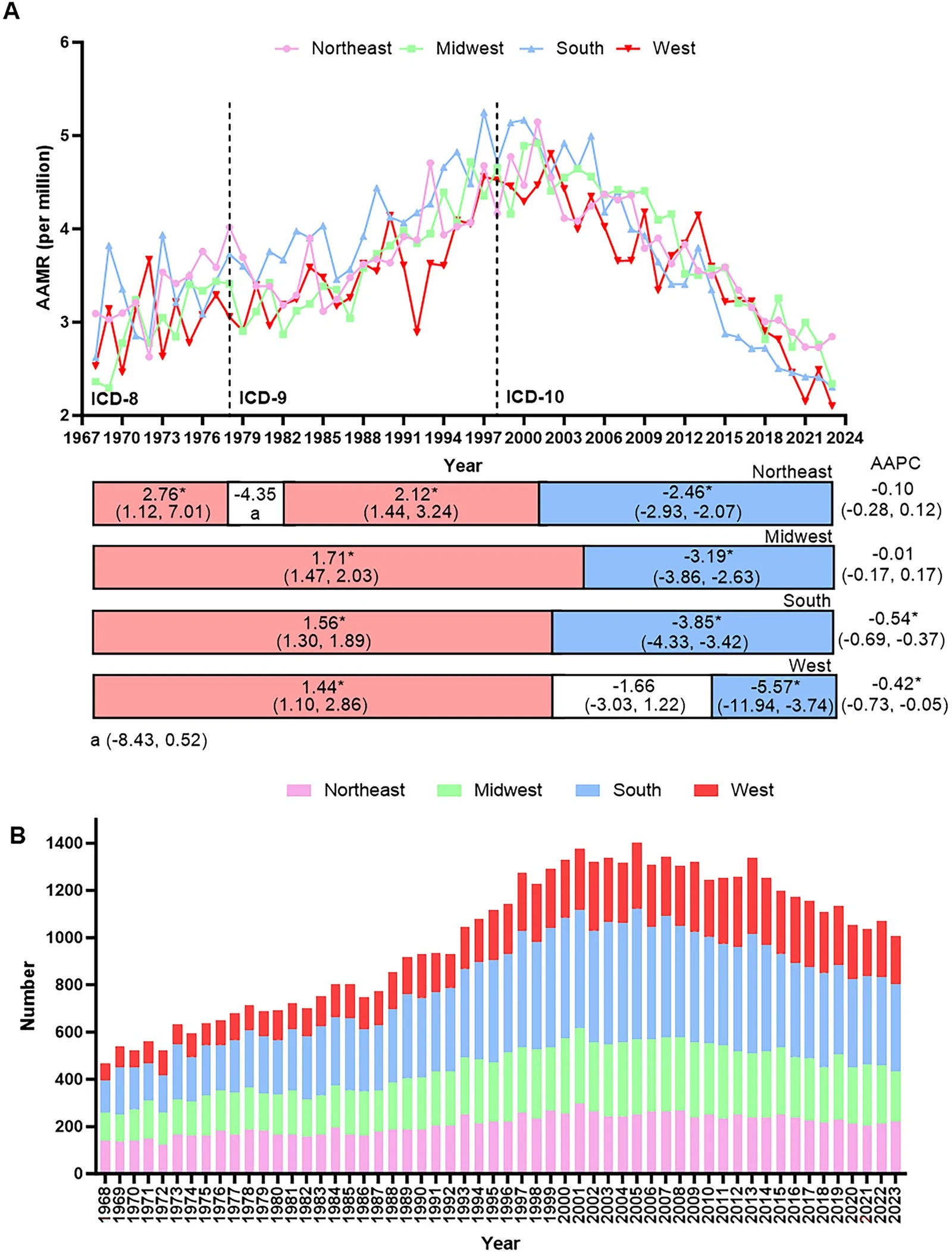
Region differences in SSc-UCD AAMR, 1968–2023. **(A)** Annual AAMR (per 1 000 000; standardized to the 2000 US standard population) by region. Vertical dashed lines indicate ICD coding eras. Segment bars show APC with 95% CI. *Indicates statistical significance. **(B)** Annual number of SSc-UCD deaths, stacked by US census region (Northeast, Midwest, South, West)

Across US census regions, the AAMR increased into the early 2000s and then decreased, with the steepest recent reduction observed in the West. Across regions, the trajectory was broadly similar but with region-specific turning points (Fig. 3; Supplementary Table S3). In the Northeast, the AAMR increased in 1968–1978 [APC 2.76% (95% CI 1.12, 7.01)], did not change significantly during 1978–1982, increased again in 1982–2001 [APC 2.12% (95% CI 1.44, 3.24)] and decreased in 2001–2023 [APC −2.46% (95% CI −2.93, −2.07)]. In the Midwest, the AAMR increased through 2004 [APC 1.71% (95% CI 1.47, 2.03)] and decreased in 2004–2023 [APC −3.19% (95% CI −3.86, −2.63)]. In the South, the AAMR increased through 2002 [APC 1.56% (95% CI 1.30, 1.89)] and decreased in 2002–2023 [APC −3.85% (95% CI −4.33, −3.42)]. In the West, the AAMR increased through 2002 [APC 1.44% (95% CI 1.10, 2.86)], showed no clear change in 2002–2014 and decreased more steeply in 2014–2023 [APC −5.57% (95% CI −11.94, −3.74)].

### Trends by age group

**Figure 4:**
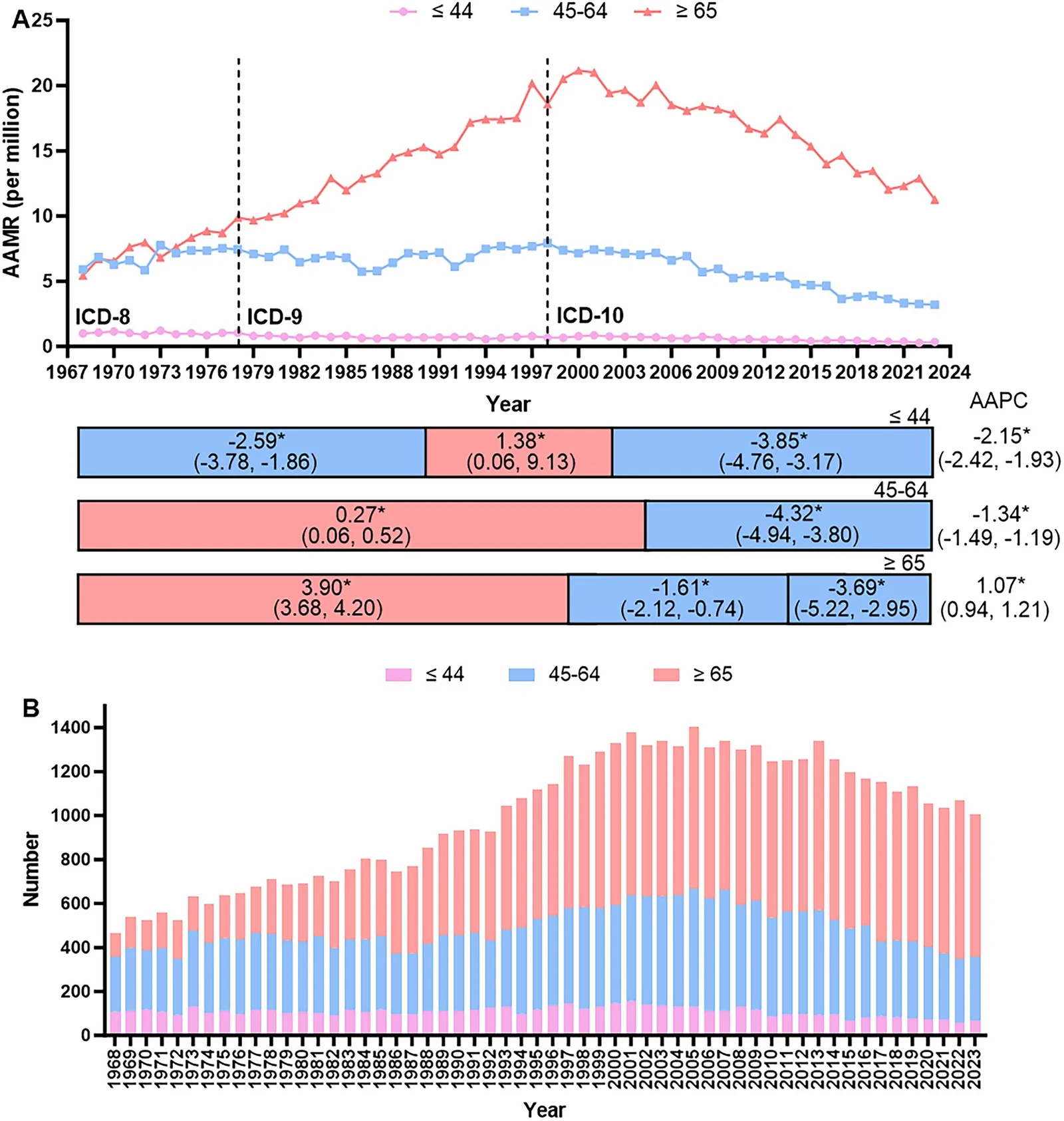
Age-group trends in SSc-UCD AAMR, 1968–2023. **(A)** Annual AAMR (per 1 000 000; standardized to the 2000 US standard population) by age group (≤44, 45–64, ≥65 years). Coloured segments below the curve show Joinpoint regression results as APC with 95% CI. *Indicates statistical significance (95% CI excluding 0). **(B)** Annual number of SSc-UCD deaths by age group

Mortality burden increasingly concentrated in older adults, and adults ≥65 years of age showed a net increase over the full period despite declines after the early-2000s peak (Fig. 4; Supplementary Table S4). In those ≤44 years of age, AAMRs decreased in 1968–1989 [APC −2.59% (95% CI −3.78, −1.86)], increased in 1989–2002 [APC 1.38% (95% CI 0.06, 9.13)] and decreased in 2002–2023 [APC −3.85% (95% CI −4.76, −3.17)], reaching 0.35 per 1 000 000 in 2023 [AAPC −2.15% (95% CI −2.42, −1.93)]. In those 45–64 years of age, rates increased up to 2004 [APC 0.27% (95% CI 0.06, 0.52)] and decreased in 2004–2023 [APC −4.32% (95% CI −4.94, −3.80)], ending at 3.20 in 2023 [AAPC −1.34% (95% CI −1.49, −1.19)]. In those ≥65 years of age, rates increased in 1968–1999 [APC 3.90% (95% CI 3.68, 4.20)], peaked around 2000–2001 (≈21 per 1 000 000) and decreased in 1999–2013 [APC −1.61% (95% CI −2.12, −0.74)] and 2013–2023 [APC −3.69% (95% CI −5.22, −2.95)]. Despite these declines, the AAMR remained 11.27 in 2023 and the long-term trend in those ≥65 years of age was upward [AAPC 1.07% (95% CI 0.94, 1.21)].

### Sensitivity analysis (MCOD), 1999–2023

**Figure 5:**
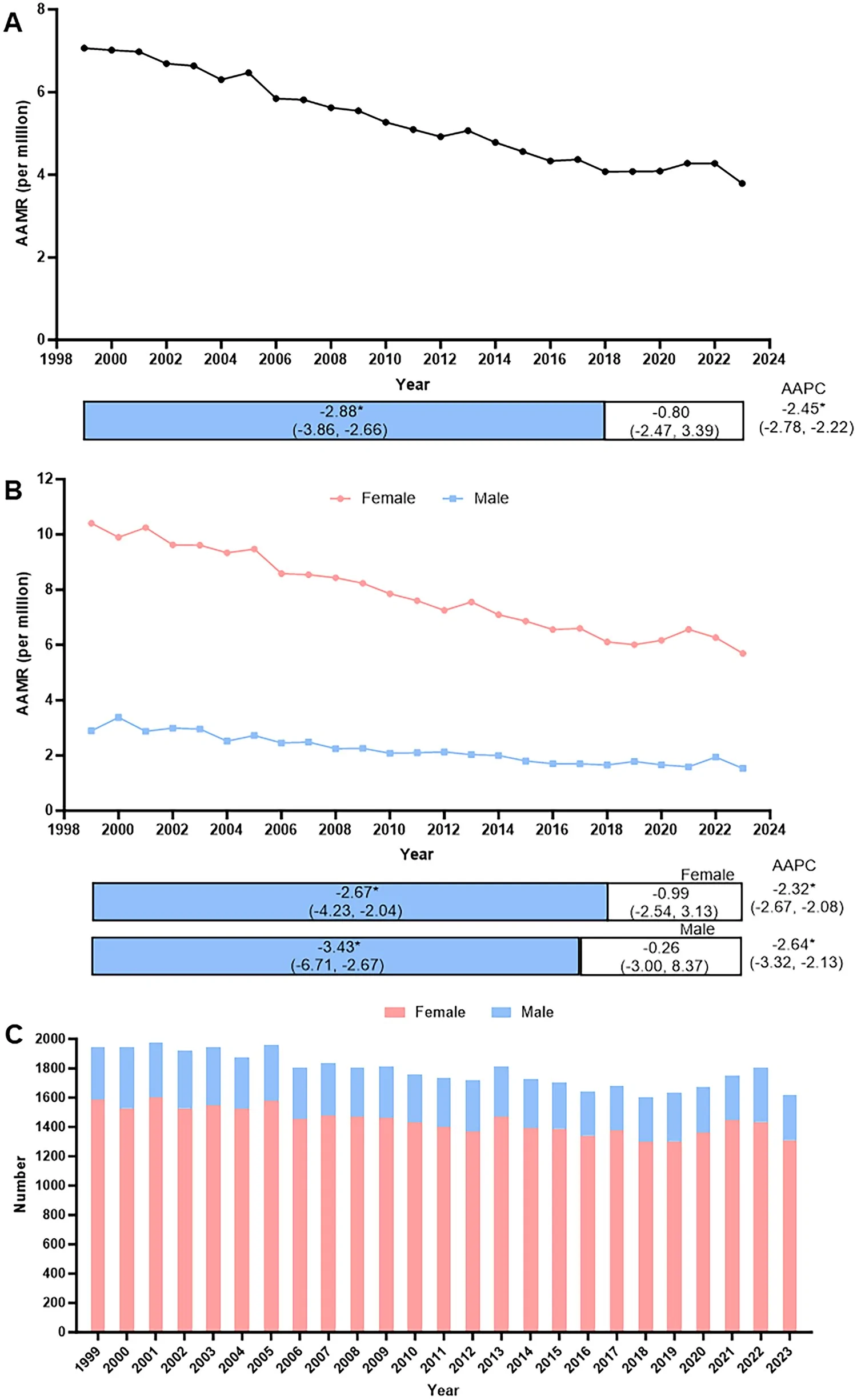
Overall and sex-specific SSc-MCOD AAMR and annual deaths, 1999–2023. **(A)** Annual age-adjusted mortality rate for SSc-MCOD. Coloured segments below the curve show Joinpoint regression results as APC with 95% CI. *Indicates statistical significance. **(B)** Sex-specific AAMR for SSc-MCOD (female and male) with APC (95% CI) displayed in the corresponding segment bars. **(C)** Annual number of SSc-MCOD deaths by sex

When SSc was recorded anywhere on the death certificate, the AAMR declined from 7.06 to 3.79 per 1 000 000, while annual deaths ranged from 1605 to 1975 (Fig. 5; Supplementary Table S5). Joinpoint regression identified a turning point around 2018, with a decrease in 1999–2018 [APC −2.88% (95% CI −3.86, −2.66)] and no clear change in 2018–2023 [APC −0.80% (95% CI −2.47, 3.39)]. The overall trend remained downward [AAPC −2.45% (95% CI −2.78, −2.22)]. Sex-stratified models showed a similar pattern. In females, rates decreased in 1999–2018 [APC −2.67% (95% CI −4.23, −2.04)] with no clear change thereafter [2018–2023; APC −0.99% (95% CI −2.54, 3.13)] and the overall 1999–2023 trend remained downward [AAPC −2.32% (95% CI −2.67, −2.08)]. In males, rates declined in 1999–2017 [APC −3.43% (95% CI −6.71, −2.67)] with no clear change in 2017–2023 [APC −0.26% (95% CI −3.00, 8.37)], while the overall decrease across 1999–2023 was significant [AAPC −2.64% (95% CI −3.32, −2.13)]. Thus MCOD analyses showed an overall downward trend in SSc-related mortality during 1999–2023, although the most recent MCOD segment did not show a statistically significant decrease.

## Discussion

In this national analysis of 1968–2023 data, SSc mortality recorded as the underlying cause of death followed a long increase, peaked in the early 2000s and then decreased. The AAMR increased from 2.66 per 1 000 000 in 1968 to 4.87 in 2001 and decreased to 2.37 in 2023, with the sharpest UCD-based decrease after 2013. The Western US census region showed the steepest recent reductions, the female:male mortality ratio continued to increase and deaths became increasingly concentrated among adults ≥65 years of age, the only age group with a net increase across the full study period. Taken together, these findings show that the overall downward trend was not uniform across demographic groups.

The long increase before the early 2000s and the subsequent decrease are unlikely to have a single explanation. The early increase may partly reflect greater recognition and recording of SSc as classification criteria, serologic testing and awareness of SSc-related organ complications became more widely used, together with limited treatment options for severe organ involvement during earlier decades. The later decrease coincided with broader changes in SSc care, including more systematic cardiopulmonary surveillance, earlier recognition of ILD and pulmonary arterial hypertension (PAH), improved supportive care and expanding organ-targeted treatment options [2, 7, 11–14]. These developments provide clinical context for the downward trend, but death certificate data cannot establish that they caused the decline. Certification and coding practices may also have shaped the observed pattern. Greater recognition of SSc could increase the likelihood that SSc was recorded or selected as the UCD, whereas attribution to proximate complications such as ILD, PAH, infection, cardiovascular disease, malignancy or renal failure could reduce the likelihood that SSc was selected as the UCD [2, 7, 11–14]. ICD transitions may also have affected comparability across eras. These factors could make UCD-based decreases appear larger if interpreted in isolation. MCOD analyses were therefore used to examine whether the overall pattern was limited to UCD assignment [6].

The MCOD findings provide context for the UCD trends. By definition, MCOD includes deaths in which SSc was selected as the UCD as well as deaths in which SSc was recorded as a contributing condition. UCD represents the subset of SSc-mentioned deaths in which SSc was selected as the underlying cause under death certificate coding rules, whereas MCOD represents the broader death certificate population in which SSc was recorded anywhere on the certificate [6]. This distinction is important in SSc, because fatal cardiopulmonary or other organ complications may be selected as the UCD even when SSc is recorded elsewhere on the certificate. MCOD mortality also declined overall from 1999 to 2023, supporting the overall downward direction observed in UCD analyses under a broader any-mention definition. However, the MCOD joinpoint occurred later, around 2018, and the final MCOD segment showed a negative but non-significant trend. Therefore, the steeper recent decline observed in the UCD-based analysis should be interpreted as specific to deaths in which SSc was selected as the underlying cause rather than extended to all deaths in which SSc was mentioned. The final MCOD segment also included the COVID-19 period, during which competing causes of death and death certification practices may have influenced any-mention mortality trends [15]. Taken together, UCD and MCOD trends should be interpreted as complementary measures that capture related but not identical aspects of SSc mortality.

Our findings extend prior US CDC WONDER analyses through 2015, which similarly showed higher SSc AAMRs in women than men and a steeper increase in women during the early decades [8]. By extending the series to 2023, we further show that female excess mortality persisted in every year and that the female:male mortality ratio continued to widen (from 2.0 in 1968 to 3.6 in 2023). The widening ratio is better understood in the context of the marked female predominance of SSc. Epidemiologic studies report an approximately 4- to 5-fold higher prevalence in women than in men [16]. This larger underlying disease burden helps explain why female AAMRs remained higher despite the steeper decline among men. In the present analysis, the widening ratio appears to reflect the larger relative decrease in male SSc-UCD AAMRs from a lower baseline, while female SSc-UCD AAMRs remained higher throughout the study period. Because the male AAMR forms the denominator of the female:male ratio, a larger relative decrease from a lower male baseline can amplify the ratio even when female mortality is also decreasing. Overall, the widening ratio appears more consistent with the larger female SSc disease burden and faster decrease in male SSc-UCD mortality from a lower baseline than with a worsening trend among women.

Consistent with prior national mortality analyses through 2015, we observed persistently higher SSc mortality in Black/African American individuals than in White individuals [8]. In this study we show that this disparity remained substantial even as the net long-term decrease was larger in Black/African American individuals. Clinical cohort studies suggest that this gap may be related, at least in part, to a higher burden of severe cardiopulmonary disease in African American patients, including more advanced ILD (worse lung function and fibrosis) and a higher prevalence of pulmonary hypertension, alongside higher overall mortality [17]. National mortality work also reports substantially higher odds of SSc-related death before age 65 years in Black *vs* White individuals, highlighting a disproportionate burden of premature mortality [18]. Socio-economic and structural factors likely contribute as well. In a matched cohort, adjustment for socio-economic covariates attenuated the race mortality association and neighbourhood income remained independently associated with mortality [17]. Because death certificate data do not capture phenotype, disease duration or access to care measures, our analysis cannot separate the contributions of disease severity from those of structural determinants.

Age-stratified analyses showed that SSc-UCD mortality has become increasingly concentrated in older adults [5]. Rates decreased over the long term in the two younger age groups, whereas the ≥65-year group showed a positive long-term trend across 1968–2023 despite decreases after the early-2000s peak. This pattern is best viewed as a historical net increase driven largely by the marked increase before 1999 rather than as evidence of a continuing recent increase among older adults. The use of age-adjusted rates also makes a simple demographic aging explanation unlikely. Overall, the pattern points to a shift in the recorded SSc mortality burden toward older ages. Improved survival to older ages is one possible contributor, but the same pattern may also reflect greater recognition or certification of SSc among older adults, changes in the number of older adults living with SSc or differences in the clinical profile of older patients [19]. Without incidence, prevalence, disease duration, longitudinal follow-up or clinical phenotype data in CDC WONDER, these mechanisms cannot be separated. Clinically, the increasing concentration of SSc-UCD deaths among older adults underscores the importance of continued follow-up and organ surveillance in older patients, while maintaining attention to younger high-risk groups [7, 10, 12]. Across US census regions, the long-term pattern was broadly similar, with rates increasing into the early 2000s and decreasing thereafter. The Western US census region showed the steepest drop in the most recent period, but the overall trajectories were largely parallel across regions.

This study benefits from complete US vital statistics over 56 years, consistent age adjustment and joinpoint regression to identify statistically supported changes in slope. By presenting both UCD and MCOD definitions and stratifying by sex, race, region and age group, these national estimates provide a practical benchmark that complements referral-centre cohorts.

### Implications for clinical practice

Persistent sex- and race-related disparities and the increasing concentration of deaths among adults ≥65 years of age indicate that attention to high-risk groups remains important despite the overall downward trend in mortality. For rheumatologists, these patterns support closer longitudinal follow-up of older patients and patients from demographic groups with higher mortality. They also reinforce the clinical importance of established cardiopulmonary screening and follow-up pathways, particularly for ILD and PAH, which remain major contributors to SSc-related mortality.

Several limitations should be considered. This study relied on death certificate data, in which both the recording of SSc and the selection of the underlying cause of death may be incomplete or inaccurate. SSc may be omitted from the certificate or recorded only as a contributing condition when death is assigned to proximate causes such as ILD, PAH, infection, malignancy or cardiovascular disease. CDC WONDER also lacks clinical information on SSc subtype, organ involvement, autoantibody status, disease duration, disease severity, treatment exposure and individual-level comorbidities. Accordingly, we could not determine the extent to which the observed trends reflected changes in the underlying SSc disease burden, patient survival, clinical phenotype, treatment exposure or cause-of-death attribution. Sparse annual counts limited modelling for racial and ethnic groups beyond Black/African American and White individuals. We also lacked individual-level socio-economic information and granular geographic measures, which limited adjustment for social determinants of health and prevented evaluation of urban–rural differences, proximity to specialist centres or local variation in access to expert SSc care. The study was not prospectively registered, reflecting its retrospective use of publicly available aggregate mortality data. Finally, ICD transitions and the years overlapping the COVID-19 period may have influenced recent estimates, although these issues are unlikely to account for the sustained multidecade pattern. COVID-19 may have affected 2020–2023 estimates through excess respiratory and infectious mortality and through changes in cause-of-death attribution, particularly when SSc was listed as a contributing condition rather than the underlying cause [15].

## Conclusion

In this national mortality analysis, SSc-attributed mortality decreased from its early-2000s peak but remained unevenly distributed by sex, race and age. Persistent disparities and the increasing concentration of deaths among older adults indicate that the burden of SSc mortality remains substantial in specific patient groups despite the overall downward trend. Further studies linking mortality data with clinical registries or cohorts may help clarify whether these patterns reflect changes in disease burden, case fatality, certification practices, access to specialist care or a combination of these factors.

## Supplementary Material

rkag107_Supplementary_Data

## Data Availability

The study used publicly available data from the CDC WONDER Multiple Cause of Death database, and the relevant case definitions, ICD-10 codes, and analytic methods are described in the manuscript and supplementary materials. No additional data repository links or identifiers are required.
