# Supplementary material for: 56-year trends and disparities in systemic sclerosis mortality in the United States, 1968–2023: a retrospective population-based study
Source: Rheumatol Adv Pract. 2026 Aug 31;10(4):rkag107. doi: 10.1093/rap/rkag107 (PMC13600682; doi:10.1093/rap/rkag107)
Supplement: Supplementary file 1 [file rkag107_supplementary_data.docx]

**Supplementary material**

Supplementary Table S1. AAMR for SSc as the underlying cause of death, by sex, United States, 1968–2023

Supplementary Table S2. AAMR for SSc as the underlying cause of death, by race, United States, 1968–2023

Supplementary Table S3. AAMR for SSc as the underlying cause of death, by U.S. census region, 1968–2023

Supplementary Table S4. AAMR for SSc as the underlying cause of death, by age groups, United States, 1968–2023

Supplementary Table S5. AAMR for SSc as the multiple cause of death, by sex, United States, 1999–2023

Supplementary Table S1. AAMR for SSc as the underlying cause of death, by sex, United States, 1968–2023

|  | Total | | | | | Female | | | | | Male | | | | |
| --- | --- | --- | --- | --- | --- | --- | --- | --- | --- | --- | --- | --- | --- | --- | --- |
| Year | Number | AAMR | 95%CI | | SE | Number | AAMR | 95%CI | | SE | Number | AAMR | 95%CI | | SE |
| 1968 | 466 | 2.66 | 2.42 | 2.91 | 0.13 | 321 | 3.48 | 3.09 | 3.86 | 0.20 | 145 | 1.77 | 1.47 | 2.07 | 0.15 |
| 1969 | 540 | 3.08 | 2.81 | 3.34 | 0.13 | 376 | 4.00 | 3.59 | 4.41 | 0.21 | 164 | 2.04 | 1.72 | 2.36 | 0.16 |
| 1970 | 524 | 2.98 | 2.72 | 3.24 | 0.13 | 382 | 4.05 | 3.64 | 4.46 | 0.21 | 142 | 1.78 | 1.48 | 2.08 | 0.15 |
| 1971 | 561 | 3.10 | 2.84 | 3.37 | 0.13 | 398 | 4.14 | 3.73 | 4.56 | 0.21 | 163 | 1.88 | 1.58 | 2.17 | 0.15 |
| 1972 | 524 | 2.89 | 2.64 | 3.14 | 0.13 | 380 | 3.90 | 3.50 | 4.30 | 0.20 | 144 | 1.67 | 1.39 | 1.95 | 0.14 |
| 1973 | 632 | 3.38 | 3.12 | 3.65 | 0.14 | 480 | 4.81 | 4.37 | 5.24 | 0.22 | 152 | 1.72 | 1.44 | 2.00 | 0.14 |
| 1974 | 596 | 3.17 | 2.91 | 3.43 | 0.13 | 445 | 4.38 | 3.96 | 4.79 | 0.21 | 151 | 1.77 | 1.48 | 2.06 | 0.15 |
| 1975 | 636 | 3.36 | 3.09 | 3.62 | 0.14 | 473 | 4.60 | 4.18 | 5.02 | 0.22 | 163 | 1.92 | 1.61 | 2.22 | 0.15 |
| 1976 | 648 | 3.31 | 3.05 | 3.57 | 0.13 | 472 | 4.44 | 4.03 | 4.85 | 0.21 | 176 | 1.96 | 1.66 | 2.26 | 0.15 |
| 1977 | 679 | 3.45 | 3.18 | 3.72 | 0.14 | 482 | 4.51 | 4.10 | 4.93 | 0.21 | 197 | 2.24 | 1.92 | 2.57 | 0.17 |
| 1978 | 713 | 3.59 | 3.32 | 3.86 | 0.14 | 543 | 5.07 | 4.63 | 5.50 | 0.22 | 170 | 1.87 | 1.58 | 2.16 | 0.15 |
| 1979 | 686 | 3.33 | 3.08 | 3.58 | 0.13 | 499 | 4.42 | 4.03 | 4.82 | 0.20 | 187 | 2.04 | 1.74 | 2.35 | 0.15 |
| 1980 | 690 | 3.33 | 3.08 | 3.58 | 0.13 | 512 | 4.52 | 4.12 | 4.92 | 0.20 | 178 | 1.87 | 1.59 | 2.15 | 0.14 |
| 1981 | 724 | 3.44 | 3.18 | 3.69 | 0.13 | 553 | 4.78 | 4.37 | 5.18 | 0.21 | 171 | 1.80 | 1.52 | 2.07 | 0.14 |
| 1982 | 703 | 3.27 | 3.02 | 3.52 | 0.13 | 547 | 4.64 | 4.25 | 5.04 | 0.20 | 156 | 1.63 | 1.37 | 1.89 | 0.13 |
| 1983 | 753 | 3.47 | 3.22 | 3.73 | 0.13 | 566 | 4.74 | 4.34 | 5.14 | 0.20 | 187 | 1.93 | 1.64 | 2.21 | 0.14 |
| 1984 | 803 | 3.66 | 3.40 | 3.91 | 0.13 | 619 | 5.13 | 4.71 | 5.54 | 0.21 | 184 | 1.89 | 1.61 | 2.17 | 0.14 |
| 1985 | 801 | 3.57 | 3.31 | 3.82 | 0.13 | 588 | 4.78 | 4.38 | 5.17 | 0.20 | 213 | 2.10 | 1.82 | 2.39 | 0.15 |
| 1986 | 746 | 3.33 | 3.09 | 3.57 | 0.12 | 559 | 4.50 | 4.12 | 4.87 | 0.19 | 187 | 1.84 | 1.57 | 2.11 | 0.14 |
| 1987 | 771 | 3.36 | 3.12 | 3.60 | 0.12 | 597 | 4.71 | 4.32 | 5.09 | 0.20 | 174 | 1.68 | 1.42 | 1.94 | 0.13 |
| 1988 | 852 | 3.72 | 3.46 | 3.97 | 0.13 | 665 | 5.21 | 4.81 | 5.61 | 0.21 | 187 | 1.87 | 1.59 | 2.15 | 0.14 |
| 1989 | 917 | 3.93 | 3.67 | 4.19 | 0.13 | 703 | 5.44 | 5.03 | 5.85 | 0.21 | 214 | 2.06 | 1.78 | 2.34 | 0.14 |
| 1990 | 932 | 3.94 | 3.68 | 4.20 | 0.13 | 706 | 5.38 | 4.98 | 5.78 | 0.21 | 226 | 2.18 | 1.89 | 2.47 | 0.15 |
| 1991 | 936 | 3.92 | 3.66 | 4.17 | 0.13 | 715 | 5.40 | 5.00 | 5.80 | 0.20 | 221 | 2.09 | 1.81 | 2.37 | 0.14 |
| 1992 | 928 | 3.78 | 3.53 | 4.02 | 0.12 | 736 | 5.39 | 5.00 | 5.79 | 0.20 | 192 | 1.76 | 1.50 | 2.01 | 0.13 |
| 1993 | 1046 | 4.17 | 3.92 | 4.43 | 0.13 | 849 | 6.09 | 5.68 | 6.51 | 0.21 | 197 | 1.76 | 1.51 | 2.01 | 0.13 |
| 1994 | 1077 | 4.24 | 3.98 | 4.49 | 0.13 | 829 | 5.89 | 5.49 | 6.30 | 0.21 | 248 | 2.17 | 1.90 | 2.44 | 0.14 |
| 1995 | 1117 | 4.34 | 4.09 | 4.60 | 0.13 | 902 | 6.35 | 5.93 | 6.77 | 0.21 | 215 | 1.89 | 1.63 | 2.14 | 0.13 |
| 1996 | 1142 | 4.37 | 4.11 | 4.62 | 0.13 | 909 | 6.31 | 5.90 | 6.72 | 0.21 | 233 | 1.99 | 1.73 | 2.25 | 0.13 |
| 1997 | 1273 | 4.78 | 4.51 | 5.04 | 0.13 | 1042 | 7.09 | 6.66 | 7.52 | 0.22 | 231 | 1.94 | 1.69 | 2.19 | 0.13 |
| 1998 | 1229 | 4.55 | 4.29 | 4.80 | 0.13 | 1003 | 6.75 | 6.33 | 7.17 | 0.21 | 226 | 1.84 | 1.60 | 2.08 | 0.12 |
| 1999 | 1291 | 4.68 | 4.42 | 4.93 | 0.13 | 1049 | 6.94 | 6.52 | 7.36 | 0.22 | 242 | 1.96 | 1.71 | 2.21 | 0.13 |
| 2000 | 1331 | 4.79 | 4.53 | 5.04 | 0.13 | 1037 | 6.77 | 6.35 | 7.18 | 0.21 | 294 | 2.33 | 2.06 | 2.60 | 0.14 |
| 2001 | 1378 | 4.87 | 4.61 | 5.13 | 0.13 | 1106 | 7.10 | 6.68 | 7.52 | 0.21 | 272 | 2.08 | 1.83 | 2.33 | 0.13 |
| 2002 | 1322 | 4.59 | 4.34 | 4.83 | 0.13 | 1035 | 6.58 | 6.18 | 6.98 | 0.21 | 287 | 2.15 | 1.90 | 2.40 | 0.13 |
| 2003 | 1339 | 4.56 | 4.32 | 4.81 | 0.12 | 1071 | 6.67 | 6.27 | 7.07 | 0.20 | 268 | 1.99 | 1.75 | 2.23 | 0.12 |
| 2004 | 1315 | 4.40 | 4.17 | 4.64 | 0.12 | 1070 | 6.57 | 6.17 | 6.96 | 0.20 | 245 | 1.75 | 1.53 | 1.98 | 0.11 |
| 2005 | 1401 | 4.59 | 4.35 | 4.84 | 0.12 | 1116 | 6.73 | 6.33 | 7.12 | 0.20 | 285 | 2.01 | 1.77 | 2.25 | 0.12 |
| 2006 | 1308 | 4.22 | 3.99 | 4.44 | 0.12 | 1043 | 6.17 | 5.80 | 6.55 | 0.19 | 265 | 1.83 | 1.61 | 2.06 | 0.11 |
| 2007 | 1341 | 4.22 | 4.00 | 4.45 | 0.12 | 1082 | 6.27 | 5.89 | 6.65 | 0.19 | 259 | 1.75 | 1.54 | 1.97 | 0.11 |
| 2008 | 1302 | 4.08 | 3.86 | 4.31 | 0.11 | 1067 | 6.13 | 5.76 | 6.50 | 0.19 | 235 | 1.56 | 1.36 | 1.77 | 0.10 |
| 2009 | 1322 | 4.06 | 3.84 | 4.28 | 0.11 | 1069 | 6.07 | 5.70 | 6.44 | 0.19 | 253 | 1.61 | 1.41 | 1.81 | 0.10 |
| 2010 | 1246 | 3.74 | 3.53 | 3.95 | 0.11 | 1014 | 5.60 | 5.25 | 5.95 | 0.18 | 232 | 1.48 | 1.29 | 1.67 | 0.10 |
| 2011 | 1252 | 3.69 | 3.48 | 3.90 | 0.11 | 1015 | 5.52 | 5.17 | 5.86 | 0.18 | 237 | 1.51 | 1.31 | 1.70 | 0.10 |
| 2012 | 1255 | 3.59 | 3.38 | 3.79 | 0.10 | 1004 | 5.34 | 5.01 | 5.68 | 0.17 | 251 | 1.52 | 1.33 | 1.71 | 0.10 |
| 2013 | 1339 | 3.74 | 3.54 | 3.94 | 0.10 | 1083 | 5.58 | 5.24 | 5.91 | 0.17 | 256 | 1.51 | 1.32 | 1.70 | 0.10 |
| 2014 | 1254 | 3.47 | 3.28 | 3.67 | 0.10 | 1001 | 5.14 | 4.81 | 5.46 | 0.17 | 253 | 1.52 | 1.33 | 1.72 | 0.10 |
| 2015 | 1197 | 3.24 | 3.06 | 3.43 | 0.10 | 970 | 4.84 | 4.53 | 5.15 | 0.16 | 227 | 1.32 | 1.14 | 1.49 | 0.09 |
| 2016 | 1170 | 3.11 | 2.93 | 3.29 | 0.09 | 956 | 4.70 | 4.39 | 5.00 | 0.16 | 214 | 1.21 | 1.04 | 1.37 | 0.08 |
| 2017 | 1154 | 2.99 | 2.82 | 3.17 | 0.09 | 941 | 4.53 | 4.23 | 4.82 | 0.15 | 213 | 1.18 | 1.02 | 1.34 | 0.08 |
| 2018 | 1108 | 2.82 | 2.65 | 2.99 | 0.09 | 889 | 4.22 | 3.94 | 4.51 | 0.15 | 219 | 1.17 | 1.01 | 1.33 | 0.08 |
| 2019 | 1135 | 2.83 | 2.66 | 3.00 | 0.09 | 903 | 4.19 | 3.91 | 4.47 | 0.14 | 232 | 1.23 | 1.07 | 1.39 | 0.08 |
| 2020 | 1052 | 2.58 | 2.42 | 2.74 | 0.08 | 863 | 3.92 | 3.66 | 4.19 | 0.14 | 189 | 1.02 | 0.87 | 1.17 | 0.08 |
| 2021 | 1036 | 2.55 | 2.39 | 2.71 | 0.08 | 849 | 3.87 | 3.61 | 4.14 | 0.14 | 187 | 0.97 | 0.83 | 1.11 | 0.07 |
| 2022 | 1069 | 2.54 | 2.39 | 2.70 | 0.08 | 859 | 3.79 | 3.53 | 4.05 | 0.13 | 210 | 1.10 | 0.95 | 1.26 | 0.08 |
| 2023 | 1005 | 2.37 | 2.22 | 2.51 | 0.08 | 808 | 3.53 | 3.28 | 3.79 | 0.13 | 197 | 0.98 | 0.84 | 1.13 | 0.07 |

AAMR: age adjusted mortality rates

Supplementary Table S2. AAMR for SSc as the underlying cause of death, by race, United States, 1968–2023

|  | Black or African American | | | | | White | | | | |
| --- | --- | --- | --- | --- | --- | --- | --- | --- | --- | --- |
| Year | Number | AAMR | 95%CI | | SE | Number | AAMR | 95%CI | | SE |
| 1968 | 80 | 4.85 | 3.83 | 6.06 | 0.55 | 378 | 2.40 | 2.15 | 2.64 | 0.12 |
| 1969 | 92 | 5.82 | 4.65 | 7.20 | 0.63 | 445 | 2.80 | 2.54 | 3.07 | 0.13 |
| 1970 | 99 | 6.28 | 5.07 | 7.69 | 0.65 | 414 | 2.59 | 2.34 | 2.84 | 0.13 |
| 1971 | 92 | 5.52 | 4.43 | 6.79 | 0.59 | 464 | 2.84 | 2.57 | 3.10 | 0.13 |
| 1972 | 90 | 5.25 | 4.21 | 6.47 | 0.56 | 430 | 2.61 | 2.36 | 2.87 | 0.13 |
| 1973 | 112 | 6.52 | 5.29 | 7.74 | 0.63 | 513 | 3.04 | 2.77 | 3.31 | 0.14 |
| 1974 | 102 | 5.70 | 4.57 | 6.82 | 0.57 | 487 | 2.87 | 2.61 | 3.13 | 0.13 |
| 1975 | 98 | 5.55 | 4.49 | 6.78 | 0.57 | 532 | 3.11 | 2.84 | 3.38 | 0.14 |
| 1976 | 100 | 5.42 | 4.34 | 6.50 | 0.55 | 537 | 3.06 | 2.79 | 3.32 | 0.13 |
| 1977 | 113 | 5.99 | 4.86 | 7.13 | 0.58 | 554 | 3.13 | 2.86 | 3.40 | 0.14 |
| 1978 | 119 | 6.48 | 5.30 | 7.66 | 0.60 | 592 | 3.31 | 3.03 | 3.58 | 0.14 |
| 1979 | 129 | 6.68 | 5.50 | 7.86 | 0.60 | 541 | 2.93 | 2.68 | 3.18 | 0.13 |
| 1980 | 120 | 6.11 | 4.99 | 7.23 | 0.57 | 563 | 3.03 | 2.77 | 3.29 | 0.13 |
| 1981 | 116 | 5.86 | 4.75 | 6.96 | 0.56 | 598 | 3.17 | 2.91 | 3.43 | 0.13 |
| 1982 | 136 | 6.75 | 5.58 | 7.91 | 0.59 | 559 | 2.88 | 2.64 | 3.13 | 0.12 |
| 1983 | 128 | 6.20 | 5.09 | 7.30 | 0.56 | 620 | 3.20 | 2.94 | 3.46 | 0.13 |
| 1984 | 145 | 6.77 | 5.64 | 7.90 | 0.58 | 647 | 3.30 | 3.04 | 3.56 | 0.13 |
| 1985 | 122 | 5.58 | 4.57 | 6.59 | 0.52 | 662 | 3.31 | 3.05 | 3.56 | 0.13 |
| 1986 | 139 | 6.44 | 5.34 | 7.54 | 0.56 | 596 | 2.99 | 2.75 | 3.24 | 0.12 |
| 1987 | 122 | 5.46 | 4.46 | 6.45 | 0.51 | 636 | 3.11 | 2.87 | 3.36 | 0.13 |
| 1988 | 125 | 5.59 | 4.59 | 6.60 | 0.51 | 704 | 3.46 | 3.20 | 3.72 | 0.13 |
| 1989 | 161 | 7.09 | 5.97 | 8.20 | 0.57 | 739 | 3.56 | 3.30 | 3.82 | 0.13 |
| 1990 | 152 | 6.52 | 5.46 | 7.58 | 0.54 | 759 | 3.62 | 3.36 | 3.88 | 0.13 |
| 1991 | 159 | 6.68 | 5.63 | 7.74 | 0.54 | 762 | 3.61 | 3.36 | 3.87 | 0.13 |
| 1992 | 165 | 6.79 | 5.73 | 7.85 | 0.54 | 749 | 3.44 | 3.20 | 3.69 | 0.13 |
| 1993 | 147 | 5.86 | 4.89 | 6.82 | 0.49 | 878 | 3.97 | 3.71 | 4.23 | 0.13 |
| 1994 | 178 | 7.09 | 6.03 | 8.14 | 0.54 | 879 | 3.91 | 3.65 | 4.17 | 0.13 |
| 1995 | 182 | 7.07 | 6.02 | 8.11 | 0.53 | 904 | 3.99 | 3.73 | 4.25 | 0.13 |
| 1996 | 189 | 6.94 | 5.93 | 7.95 | 0.51 | 934 | 4.08 | 3.82 | 4.34 | 0.13 |
| 1997 | 232 | 8.46 | 7.35 | 9.57 | 0.57 | 1009 | 4.33 | 4.06 | 4.60 | 0.14 |
| 1998 | 172 | 6.10 | 5.18 | 7.03 | 0.47 | 1031 | 4.37 | 4.11 | 4.64 | 0.14 |
| 1999 | 215 | 7.46 | 6.45 | 8.47 | 0.52 | 1043 | 4.35 | 4.09 | 4.62 | 0.14 |
| 2000 | 231 | 7.77 | 6.75 | 8.79 | 0.52 | 1063 | 4.40 | 4.14 | 4.67 | 0.14 |
| 2001 | 205 | 6.72 | 5.79 | 7.66 | 0.48 | 1139 | 4.63 | 4.37 | 4.90 | 0.14 |
| 2002 | 201 | 6.62 | 5.69 | 7.55 | 0.48 | 1090 | 4.38 | 4.12 | 4.64 | 0.13 |
| 2003 | 217 | 6.73 | 5.82 | 7.65 | 0.47 | 1085 | 4.30 | 4.04 | 4.56 | 0.13 |
| 2004 | 191 | 5.88 | 5.03 | 6.74 | 0.43 | 1081 | 4.21 | 3.96 | 4.47 | 0.13 |
| 2005 | 234 | 7.00 | 6.08 | 7.91 | 0.47 | 1124 | 4.29 | 4.04 | 4.54 | 0.13 |
| 2006 | 217 | 6.29 | 5.44 | 7.15 | 0.44 | 1054 | 3.96 | 3.72 | 4.20 | 0.12 |
| 2007 | 205 | 5.63 | 4.84 | 6.41 | 0.40 | 1098 | 4.06 | 3.82 | 4.31 | 0.12 |
| 2008 | 213 | 5.99 | 5.17 | 6.81 | 0.42 | 1051 | 3.88 | 3.64 | 4.12 | 0.12 |
| 2009 | 223 | 5.91 | 5.11 | 6.70 | 0.40 | 1055 | 3.80 | 3.57 | 4.04 | 0.12 |
| 2010 | 205 | 5.54 | 4.76 | 6.32 | 0.40 | 997 | 3.51 | 3.29 | 3.73 | 0.11 |
| 2011 | 187 | 4.95 | 4.22 | 5.68 | 0.37 | 1024 | 3.55 | 3.33 | 3.77 | 0.11 |
| 2012 | 202 | 4.88 | 4.19 | 5.56 | 0.35 | 1010 | 3.46 | 3.24 | 3.68 | 0.11 |
| 2013 | 210 | 5.13 | 4.42 | 5.84 | 0.36 | 1071 | 3.54 | 3.32 | 3.75 | 0.11 |
| 2014 | 203 | 4.97 | 4.27 | 5.68 | 0.36 | 998 | 3.29 | 3.08 | 3.50 | 0.11 |
| 2015 | 166 | 3.94 | 3.33 | 4.56 | 0.31 | 989 | 3.17 | 2.97 | 3.38 | 0.10 |
| 2016 | 200 | 4.55 | 3.91 | 5.19 | 0.33 | 927 | 2.92 | 2.73 | 3.12 | 0.10 |
| 2017 | 195 | 4.63 | 3.96 | 5.29 | 0.34 | 916 | 2.81 | 2.62 | 3.00 | 0.09 |
| 2018 | 195 | 4.51 | 3.86 | 5.16 | 0.33 | 864 | 2.61 | 2.43 | 2.79 | 0.09 |
| 2019 | 169 | 3.66 | 3.10 | 4.23 | 0.29 | 923 | 2.76 | 2.58 | 2.94 | 0.09 |
| 2020 | 175 | 3.74 | 3.18 | 4.31 | 0.29 | 827 | 2.43 | 2.26 | 2.60 | 0.09 |
| 2021 | 157 | 3.39 | 2.85 | 3.93 | 0.28 | 837 | 2.50 | 2.32 | 2.67 | 0.09 |
| 2022 | 164 | 3.69 | 3.11 | 4.26 | 0.29 | 853 | 2.45 | 2.28 | 2.62 | 0.09 |
| 2023 | 157 | 3.31 | 2.78 | 3.84 | 0.27 | 788 | 2.25 | 2.09 | 2.41 | 0.08 |

AAMR: age adjusted mortality rates

Supplementary Table S3. AAMR for SSc as the underlying cause of death, by U.S. census region, 1968–2023

|  | Northeast | | | | | Midwest | | | | | South | | | | | West | | | | |
| --- | --- | --- | --- | --- | --- | --- | --- | --- | --- | --- | --- | --- | --- | --- | --- | --- | --- | --- | --- | --- |
| Year | Number | AAMR | 95%CI | | SE | Number | AAMR | 95%CI | | SE | Number | AAMR | 95%CI | | SE | Number | AAMR | 95%CI | | SE |
| 1968 | 140 | 3.09 | 2.57 | 3.61 | 0.27 | 117 | 2.36 | 1.93 | 2.80 | 0.22 | 139 | 2.62 | 2.18 | 3.06 | 0.23 | 70 | 2.53 | 1.96 | 3.21 | 0.31 |
| 1969 | 137 | 3.03 | 2.51 | 3.55 | 0.26 | 114 | 2.30 | 1.87 | 2.73 | 0.22 | 199 | 3.82 | 3.28 | 4.36 | 0.27 | 90 | 3.14 | 2.52 | 3.87 | 0.34 |
| 1970 | 140 | 3.10 | 2.57 | 3.62 | 0.27 | 134 | 2.77 | 2.30 | 3.25 | 0.24 | 177 | 3.36 | 2.85 | 3.86 | 0.26 | 73 | 2.47 | 1.93 | 3.10 | 0.29 |
| 1971 | 149 | 3.21 | 2.69 | 3.74 | 0.27 | 160 | 3.24 | 2.73 | 3.75 | 0.26 | 159 | 2.86 | 2.40 | 3.31 | 0.23 | 93 | 3.14 | 2.53 | 3.86 | 0.33 |
| 1972 | 122 | 2.63 | 2.15 | 3.10 | 0.24 | 136 | 2.77 | 2.30 | 3.25 | 0.24 | 158 | 2.78 | 2.34 | 3.23 | 0.23 | 108 | 3.67 | 2.96 | 4.38 | 0.36 |
| 1973 | 164 | 3.54 | 2.99 | 4.09 | 0.28 | 150 | 3.05 | 2.55 | 3.55 | 0.25 | 233 | 3.94 | 3.42 | 4.45 | 0.26 | 85 | 2.63 | 2.10 | 3.26 | 0.29 |
| 1974 | 163 | 3.41 | 2.88 | 3.95 | 0.27 | 142 | 2.84 | 2.37 | 3.32 | 0.24 | 189 | 3.21 | 2.75 | 3.68 | 0.24 | 102 | 3.22 | 2.58 | 3.85 | 0.32 |
| 1975 | 161 | 3.49 | 2.94 | 4.05 | 0.28 | 169 | 3.40 | 2.88 | 3.92 | 0.27 | 215 | 3.51 | 3.03 | 3.99 | 0.24 | 91 | 2.78 | 2.23 | 3.42 | 0.30 |
| 1976 | 181 | 3.76 | 3.20 | 4.32 | 0.29 | 170 | 3.34 | 2.83 | 3.85 | 0.26 | 194 | 3.09 | 2.65 | 3.53 | 0.23 | 103 | 3.08 | 2.47 | 3.68 | 0.31 |
| 1977 | 168 | 3.59 | 3.03 | 4.15 | 0.28 | 176 | 3.44 | 2.92 | 3.96 | 0.26 | 223 | 3.44 | 2.98 | 3.91 | 0.24 | 112 | 3.29 | 2.67 | 3.91 | 0.32 |
| 1978 | 189 | 4.02 | 3.43 | 4.60 | 0.30 | 176 | 3.41 | 2.90 | 3.93 | 0.26 | 242 | 3.73 | 3.25 | 4.21 | 0.24 | 106 | 3.06 | 2.47 | 3.65 | 0.30 |
| 1979 | 184 | 3.70 | 3.15 | 4.24 | 0.28 | 154 | 2.91 | 2.44 | 3.37 | 0.24 | 242 | 3.60 | 3.14 | 4.07 | 0.24 | 106 | 2.91 | 2.34 | 3.47 | 0.29 |
| 1980 | 166 | 3.39 | 2.86 | 3.91 | 0.27 | 169 | 3.11 | 2.63 | 3.59 | 0.24 | 232 | 3.41 | 2.96 | 3.86 | 0.23 | 123 | 3.40 | 2.79 | 4.01 | 0.31 |
| 1981 | 166 | 3.38 | 2.86 | 3.91 | 0.27 | 187 | 3.42 | 2.92 | 3.93 | 0.26 | 257 | 3.76 | 3.29 | 4.23 | 0.24 | 114 | 2.96 | 2.41 | 3.52 | 0.28 |
| 1982 | 159 | 3.18 | 2.68 | 3.69 | 0.26 | 156 | 2.87 | 2.41 | 3.33 | 0.23 | 265 | 3.67 | 3.22 | 4.12 | 0.23 | 123 | 3.19 | 2.61 | 3.76 | 0.29 |
| 1983 | 164 | 3.29 | 2.78 | 3.80 | 0.26 | 169 | 3.12 | 2.64 | 3.60 | 0.24 | 290 | 3.98 | 3.51 | 4.45 | 0.24 | 130 | 3.25 | 2.68 | 3.81 | 0.29 |
| 1984 | 195 | 3.90 | 3.34 | 4.46 | 0.28 | 178 | 3.20 | 2.72 | 3.67 | 0.24 | 288 | 3.90 | 3.44 | 4.36 | 0.23 | 142 | 3.59 | 2.99 | 4.19 | 0.31 |
| 1985 | 166 | 3.12 | 2.64 | 3.60 | 0.25 | 188 | 3.38 | 2.89 | 3.88 | 0.25 | 305 | 4.04 | 3.58 | 4.50 | 0.23 | 142 | 3.48 | 2.90 | 4.06 | 0.30 |
| 1986 | 161 | 3.24 | 2.73 | 3.75 | 0.26 | 189 | 3.35 | 2.87 | 3.83 | 0.25 | 262 | 3.46 | 3.03 | 3.89 | 0.22 | 134 | 3.17 | 2.63 | 3.72 | 0.28 |
| 1987 | 179 | 3.48 | 2.96 | 4.00 | 0.26 | 173 | 3.04 | 2.58 | 3.50 | 0.23 | 275 | 3.57 | 3.14 | 3.99 | 0.22 | 144 | 3.26 | 2.72 | 3.80 | 0.28 |
| 1988 | 185 | 3.62 | 3.09 | 4.15 | 0.27 | 201 | 3.58 | 3.08 | 4.08 | 0.26 | 309 | 3.92 | 3.48 | 4.37 | 0.23 | 157 | 3.63 | 3.05 | 4.20 | 0.29 |
| 1989 | 189 | 3.67 | 3.14 | 4.20 | 0.27 | 216 | 3.73 | 3.23 | 4.24 | 0.26 | 354 | 4.44 | 3.97 | 4.91 | 0.24 | 158 | 3.55 | 2.99 | 4.11 | 0.29 |
| 1990 | 186 | 3.64 | 3.11 | 4.17 | 0.27 | 222 | 3.82 | 3.31 | 4.33 | 0.26 | 337 | 4.13 | 3.68 | 4.57 | 0.23 | 187 | 4.14 | 3.54 | 4.74 | 0.31 |
| 1991 | 204 | 3.92 | 3.37 | 4.46 | 0.28 | 230 | 3.98 | 3.46 | 4.50 | 0.26 | 336 | 4.07 | 3.63 | 4.51 | 0.22 | 166 | 3.61 | 3.06 | 4.16 | 0.28 |
| 1992 | 204 | 3.88 | 3.35 | 4.42 | 0.27 | 229 | 3.85 | 3.35 | 4.35 | 0.26 | 355 | 4.18 | 3.74 | 4.61 | 0.22 | 140 | 2.89 | 2.41 | 3.37 | 0.25 |
| 1993 | 253 | 4.71 | 4.12 | 5.29 | 0.30 | 240 | 3.95 | 3.45 | 4.45 | 0.26 | 375 | 4.27 | 3.84 | 4.70 | 0.22 | 178 | 3.63 | 3.09 | 4.16 | 0.27 |
| 1994 | 214 | 3.94 | 3.41 | 4.47 | 0.27 | 269 | 4.39 | 3.87 | 4.92 | 0.27 | 414 | 4.66 | 4.21 | 5.11 | 0.23 | 180 | 3.61 | 3.08 | 4.14 | 0.27 |
| 1995 | 219 | 4.02 | 3.49 | 4.56 | 0.27 | 251 | 4.07 | 3.56 | 4.57 | 0.26 | 435 | 4.83 | 4.37 | 5.28 | 0.23 | 212 | 4.09 | 3.54 | 4.65 | 0.28 |
| 1996 | 221 | 4.07 | 3.53 | 4.60 | 0.27 | 293 | 4.72 | 4.18 | 5.26 | 0.28 | 414 | 4.49 | 4.05 | 4.92 | 0.22 | 214 | 4.05 | 3.51 | 4.60 | 0.28 |
| 1997 | 258 | 4.68 | 4.11 | 5.25 | 0.29 | 275 | 4.36 | 3.84 | 4.87 | 0.26 | 494 | 5.25 | 4.79 | 5.71 | 0.24 | 246 | 4.56 | 3.99 | 5.13 | 0.29 |
| 1998 | 232 | 4.17 | 3.63 | 4.70 | 0.27 | 295 | 4.65 | 4.12 | 5.18 | 0.27 | 453 | 4.70 | 4.27 | 5.14 | 0.22 | 249 | 4.52 | 3.96 | 5.09 | 0.29 |
| 1999 | 269 | 4.77 | 4.20 | 5.35 | 0.29 | 266 | 4.16 | 3.66 | 4.66 | 0.26 | 505 | 5.14 | 4.69 | 5.59 | 0.23 | 251 | 4.46 | 3.91 | 5.01 | 0.28 |
| 2000 | 255 | 4.47 | 3.92 | 5.02 | 0.28 | 317 | 4.89 | 4.35 | 5.43 | 0.27 | 513 | 5.17 | 4.72 | 5.62 | 0.23 | 246 | 4.29 | 3.75 | 4.83 | 0.27 |
| 2001 | 296 | 5.15 | 4.56 | 5.73 | 0.30 | 321 | 4.92 | 4.38 | 5.46 | 0.27 | 498 | 4.93 | 4.50 | 5.37 | 0.22 | 263 | 4.47 | 3.93 | 5.01 | 0.28 |
| 2002 | 263 | 4.55 | 4.00 | 5.10 | 0.28 | 291 | 4.41 | 3.90 | 4.91 | 0.26 | 476 | 4.59 | 4.18 | 5.01 | 0.21 | 292 | 4.81 | 4.25 | 5.36 | 0.28 |
| 2003 | 244 | 4.11 | 3.60 | 4.63 | 0.26 | 304 | 4.55 | 4.04 | 5.06 | 0.26 | 519 | 4.92 | 4.49 | 5.34 | 0.22 | 272 | 4.43 | 3.90 | 4.96 | 0.27 |
| 2004 | 243 | 4.08 | 3.57 | 4.60 | 0.26 | 314 | 4.65 | 4.13 | 5.16 | 0.26 | 505 | 4.65 | 4.24 | 5.05 | 0.21 | 253 | 3.99 | 3.50 | 4.49 | 0.25 |
| 2005 | 253 | 4.25 | 3.72 | 4.77 | 0.27 | 316 | 4.56 | 4.05 | 5.06 | 0.26 | 554 | 5.00 | 4.58 | 5.41 | 0.21 | 278 | 4.35 | 3.83 | 4.86 | 0.26 |
| 2006 | 265 | 4.37 | 3.84 | 4.90 | 0.27 | 304 | 4.36 | 3.87 | 4.86 | 0.25 | 475 | 4.18 | 3.80 | 4.56 | 0.19 | 264 | 4.02 | 3.53 | 4.51 | 0.25 |
| 2007 | 263 | 4.31 | 3.79 | 4.84 | 0.27 | 315 | 4.42 | 3.93 | 4.91 | 0.25 | 514 | 4.41 | 4.02 | 4.79 | 0.20 | 249 | 3.66 | 3.20 | 4.11 | 0.23 |
| 2008 | 266 | 4.36 | 3.83 | 4.89 | 0.27 | 312 | 4.38 | 3.89 | 4.87 | 0.25 | 471 | 4.00 | 3.63 | 4.36 | 0.19 | 253 | 3.66 | 3.21 | 4.12 | 0.23 |
| 2009 | 236 | 3.79 | 3.30 | 4.28 | 0.25 | 319 | 4.41 | 3.92 | 4.89 | 0.25 | 470 | 3.93 | 3.57 | 4.29 | 0.18 | 297 | 4.18 | 3.70 | 4.66 | 0.24 |
| 2010 | 249 | 3.90 | 3.41 | 4.39 | 0.25 | 303 | 4.10 | 3.63 | 4.57 | 0.24 | 452 | 3.65 | 3.31 | 3.99 | 0.17 | 242 | 3.34 | 2.92 | 3.77 | 0.22 |
| 2011 | 235 | 3.63 | 3.16 | 4.10 | 0.24 | 308 | 4.16 | 3.69 | 4.63 | 0.24 | 431 | 3.41 | 3.08 | 3.73 | 0.17 | 278 | 3.71 | 3.27 | 4.15 | 0.23 |
| 2012 | 249 | 3.83 | 3.35 | 4.32 | 0.25 | 268 | 3.52 | 3.09 | 3.94 | 0.22 | 442 | 3.41 | 3.08 | 3.73 | 0.16 | 296 | 3.84 | 3.40 | 4.29 | 0.23 |
| 2013 | 236 | 3.55 | 3.09 | 4.01 | 0.24 | 274 | 3.50 | 3.08 | 3.93 | 0.22 | 504 | 3.80 | 3.46 | 4.13 | 0.17 | 325 | 4.15 | 3.69 | 4.61 | 0.23 |
| 2014 | 239 | 3.50 | 3.05 | 3.95 | 0.23 | 280 | 3.58 | 3.15 | 4.00 | 0.22 | 448 | 3.35 | 3.03 | 3.66 | 0.16 | 287 | 3.60 | 3.18 | 4.02 | 0.22 |
| 2015 | 251 | 3.59 | 3.14 | 4.04 | 0.23 | 284 | 3.58 | 3.16 | 4.01 | 0.22 | 397 | 2.88 | 2.59 | 3.16 | 0.15 | 265 | 3.22 | 2.82 | 3.61 | 0.20 |
| 2016 | 236 | 3.35 | 2.91 | 3.78 | 0.22 | 259 | 3.20 | 2.80 | 3.60 | 0.20 | 397 | 2.84 | 2.56 | 3.13 | 0.15 | 278 | 3.23 | 2.84 | 3.62 | 0.20 |
| 2017 | 226 | 3.16 | 2.74 | 3.58 | 0.22 | 264 | 3.17 | 2.78 | 3.56 | 0.20 | 385 | 2.72 | 2.44 | 3.00 | 0.14 | 279 | 3.23 | 2.84 | 3.61 | 0.20 |
| 2018 | 216 | 3.01 | 2.59 | 3.42 | 0.21 | 236 | 2.82 | 2.45 | 3.19 | 0.19 | 399 | 2.72 | 2.45 | 3.00 | 0.14 | 257 | 2.90 | 2.54 | 3.26 | 0.18 |
| 2019 | 228 | 3.02 | 2.62 | 3.42 | 0.20 | 278 | 3.26 | 2.87 | 3.65 | 0.20 | 376 | 2.51 | 2.25 | 2.77 | 0.13 | 253 | 2.82 | 2.46 | 3.17 | 0.18 |
| 2020 | 213 | 2.89 | 2.50 | 3.29 | 0.20 | 235 | 2.74 | 2.37 | 3.10 | 0.18 | 377 | 2.46 | 2.21 | 2.72 | 0.13 | 227 | 2.46 | 2.13 | 2.79 | 0.17 |
| 2021 | 204 | 2.73 | 2.35 | 3.12 | 0.20 | 259 | 3.00 | 2.62 | 3.37 | 0.19 | 373 | 2.42 | 2.16 | 2.67 | 0.13 | 200 | 2.15 | 1.85 | 2.45 | 0.16 |
| 2022 | 211 | 2.73 | 2.35 | 3.11 | 0.19 | 247 | 2.76 | 2.41 | 3.12 | 0.18 | 374 | 2.41 | 2.16 | 2.66 | 0.13 | 237 | 2.49 | 2.17 | 2.81 | 0.16 |
| 2023 | 221 | 2.85 | 2.46 | 3.23 | 0.20 | 213 | 2.34 | 2.02 | 2.66 | 0.16 | 369 | 2.31 | 2.06 | 2.55 | 0.12 | 202 | 2.10 | 1.81 | 2.40 | 0.15 |

AAMR: age adjusted mortality rates

Supplementary Table S4. AAMR for SSc as the underlying cause of death, by age groups, United States, 1968–2023

|  | ≤44 years | | | | | 45-64 years | | | | | ≥65 years | | | | |
| --- | --- | --- | --- | --- | --- | --- | --- | --- | --- | --- | --- | --- | --- | --- | --- |
| Year | Number | AAMR | 95%CI | | SE | Number | AAMR | 95%CI | | SE | Number | AAMR | 95%CI | | SE |
| 1968 | 109 | 1.01 | 0.82 | 1.21 | 0.10 | 246 | 5.92 | 5.17 | 6.66 | 0.38 | 111 | 5.44 | 4.41 | 6.47 | 0.53 |
| 1969 | 111 | 1.08 | 0.87 | 1.28 | 0.10 | 287 | 6.87 | 6.07 | 7.67 | 0.41 | 142 | 6.73 | 5.60 | 7.85 | 0.57 |
| 1970 | 119 | 1.16 | 0.95 | 1.37 | 0.11 | 268 | 6.28 | 5.52 | 7.03 | 0.39 | 137 | 6.56 | 5.44 | 7.68 | 0.57 |
| 1971 | 109 | 1.04 | 0.84 | 1.23 | 0.10 | 287 | 6.60 | 5.83 | 7.36 | 0.39 | 165 | 7.63 | 6.44 | 8.82 | 0.61 |
| 1972 | 94 | 0.90 | 0.72 | 1.10 | 0.09 | 254 | 5.85 | 5.13 | 6.57 | 0.37 | 176 | 7.97 | 6.77 | 9.18 | 0.62 |
| 1973 | 132 | 1.22 | 1.01 | 1.44 | 0.11 | 342 | 7.76 | 6.93 | 8.59 | 0.42 | 158 | 6.84 | 5.76 | 7.92 | 0.55 |
| 1974 | 102 | 0.95 | 0.76 | 1.13 | 0.10 | 318 | 7.15 | 6.36 | 7.94 | 0.40 | 176 | 7.62 | 6.48 | 8.76 | 0.58 |
| 1975 | 110 | 1.02 | 0.82 | 1.22 | 0.10 | 332 | 7.37 | 6.57 | 8.17 | 0.41 | 194 | 8.35 | 7.16 | 9.55 | 0.61 |
| 1976 | 98 | 0.86 | 0.70 | 1.06 | 0.09 | 336 | 7.34 | 6.55 | 8.13 | 0.40 | 214 | 8.87 | 7.66 | 10.08 | 0.62 |
| 1977 | 117 | 1.04 | 0.84 | 1.23 | 0.10 | 347 | 7.54 | 6.73 | 8.34 | 0.41 | 214 | 8.71 | 7.53 | 9.90 | 0.61 |
| 1978 | 117 | 1.05 | 0.86 | 1.25 | 0.10 | 342 | 7.46 | 6.66 | 8.26 | 0.41 | 254 | 9.89 | 8.66 | 11.12 | 0.63 |
| 1979 | 101 | 0.81 | 0.65 | 0.97 | 0.08 | 331 | 7.10 | 6.32 | 7.88 | 0.40 | 254 | 9.68 | 8.48 | 10.89 | 0.62 |
| 1980 | 106 | 0.84 | 0.68 | 1.01 | 0.08 | 319 | 6.86 | 6.09 | 7.62 | 0.39 | 265 | 9.97 | 8.75 | 11.19 | 0.62 |
| 1981 | 100 | 0.77 | 0.61 | 0.92 | 0.08 | 350 | 7.42 | 6.63 | 8.22 | 0.40 | 274 | 10.21 | 8.99 | 11.44 | 0.63 |
| 1982 | 91 | 0.68 | 0.55 | 0.84 | 0.07 | 308 | 6.47 | 5.73 | 7.21 | 0.38 | 304 | 10.99 | 9.74 | 12.24 | 0.64 |
| 1983 | 116 | 0.84 | 0.68 | 1.00 | 0.08 | 320 | 6.77 | 6.01 | 7.52 | 0.39 | 317 | 11.25 | 10.00 | 12.50 | 0.64 |
| 1984 | 104 | 0.73 | 0.59 | 0.87 | 0.07 | 333 | 6.97 | 6.20 | 7.73 | 0.39 | 366 | 12.92 | 11.58 | 14.26 | 0.68 |
| 1985 | 118 | 0.82 | 0.67 | 0.97 | 0.08 | 331 | 6.82 | 6.07 | 7.57 | 0.38 | 352 | 11.99 | 10.73 | 13.25 | 0.65 |
| 1986 | 98 | 0.65 | 0.53 | 0.79 | 0.07 | 273 | 5.75 | 5.06 | 6.45 | 0.36 | 375 | 12.89 | 11.58 | 14.21 | 0.67 |
| 1987 | 96 | 0.61 | 0.49 | 0.74 | 0.06 | 277 | 5.79 | 5.10 | 6.49 | 0.35 | 398 | 13.28 | 11.97 | 14.60 | 0.67 |
| 1988 | 110 | 0.70 | 0.57 | 0.83 | 0.07 | 306 | 6.42 | 5.69 | 7.15 | 0.37 | 436 | 14.52 | 13.14 | 15.89 | 0.70 |
| 1989 | 112 | 0.70 | 0.57 | 0.83 | 0.07 | 344 | 7.15 | 6.39 | 7.91 | 0.39 | 461 | 14.90 | 13.53 | 16.27 | 0.70 |
| 1990 | 113 | 0.69 | 0.56 | 0.82 | 0.07 | 341 | 7.02 | 6.27 | 7.77 | 0.38 | 478 | 15.28 | 13.90 | 16.66 | 0.70 |
| 1991 | 116 | 0.69 | 0.57 | 0.82 | 0.06 | 349 | 7.21 | 6.45 | 7.97 | 0.39 | 471 | 14.75 | 13.41 | 16.09 | 0.68 |
| 1992 | 124 | 0.74 | 0.61 | 0.87 | 0.07 | 305 | 6.13 | 5.44 | 6.82 | 0.35 | 499 | 15.31 | 13.96 | 16.66 | 0.69 |
| 1993 | 131 | 0.75 | 0.62 | 0.88 | 0.07 | 348 | 6.82 | 6.10 | 7.54 | 0.37 | 567 | 17.19 | 15.77 | 18.61 | 0.73 |
| 1994 | 99 | 0.57 | 0.46 | 0.69 | 0.06 | 392 | 7.49 | 6.75 | 8.23 | 0.38 | 586 | 17.43 | 16.02 | 18.85 | 0.72 |
| 1995 | 118 | 0.66 | 0.54 | 0.78 | 0.06 | 410 | 7.70 | 6.95 | 8.44 | 0.38 | 589 | 17.42 | 16.01 | 18.83 | 0.72 |
| 1996 | 135 | 0.75 | 0.62 | 0.88 | 0.06 | 408 | 7.48 | 6.75 | 8.20 | 0.37 | 599 | 17.54 | 16.13 | 18.94 | 0.72 |
| 1997 | 146 | 0.80 | 0.67 | 0.93 | 0.07 | 432 | 7.68 | 6.95 | 8.40 | 0.37 | 695 | 20.18 | 18.68 | 21.68 | 0.77 |
| 1998 | 123 | 0.67 | 0.55 | 0.79 | 0.06 | 462 | 7.92 | 7.20 | 8.64 | 0.37 | 644 | 18.58 | 17.15 | 20.02 | 0.73 |
| 1999 | 130 | 0.68 | 0.57 | 0.80 | 0.06 | 447 | 7.38 | 6.70 | 8.07 | 0.35 | 714 | 20.52 | 19.02 | 22.03 | 0.77 |
| 2000 | 148 | 0.80 | 0.67 | 0.93 | 0.07 | 443 | 7.15 | 6.49 | 7.82 | 0.34 | 740 | 21.17 | 19.64 | 22.69 | 0.78 |
| 2001 | 158 | 0.87 | 0.73 | 1.00 | 0.07 | 477 | 7.42 | 6.75 | 8.08 | 0.34 | 743 | 21.02 | 19.51 | 22.53 | 0.77 |
| 2002 | 140 | 0.77 | 0.64 | 0.90 | 0.07 | 491 | 7.32 | 6.67 | 7.97 | 0.33 | 691 | 19.43 | 17.98 | 20.88 | 0.74 |
| 2003 | 136 | 0.76 | 0.63 | 0.88 | 0.07 | 498 | 7.14 | 6.51 | 7.77 | 0.32 | 705 | 19.67 | 18.22 | 21.12 | 0.74 |
| 2004 | 131 | 0.72 | 0.60 | 0.85 | 0.06 | 506 | 7.04 | 6.43 | 7.66 | 0.31 | 678 | 18.73 | 17.32 | 20.14 | 0.72 |
| 2005 | 129 | 0.72 | 0.59 | 0.84 | 0.06 | 537 | 7.18 | 6.57 | 7.79 | 0.31 | 735 | 20.05 | 18.60 | 21.50 | 0.74 |
| 2006 | 113 | 0.62 | 0.51 | 0.74 | 0.06 | 507 | 6.60 | 6.03 | 7.18 | 0.29 | 688 | 18.53 | 17.14 | 19.91 | 0.71 |
| 2007 | 112 | 0.61 | 0.50 | 0.73 | 0.06 | 548 | 6.93 | 6.34 | 7.51 | 0.30 | 681 | 18.09 | 16.73 | 19.45 | 0.69 |
| 2008 | 130 | 0.74 | 0.61 | 0.87 | 0.07 | 462 | 5.71 | 5.19 | 6.24 | 0.27 | 710 | 18.44 | 17.08 | 19.80 | 0.69 |
| 2009 | 119 | 0.68 | 0.55 | 0.80 | 0.06 | 492 | 5.96 | 5.43 | 6.49 | 0.27 | 711 | 18.20 | 16.86 | 19.54 | 0.68 |
| 2010 | 88 | 0.49 | 0.39 | 0.60 | 0.05 | 447 | 5.25 | 4.76 | 5.74 | 0.25 | 711 | 17.86 | 16.55 | 19.18 | 0.67 |
| 2011 | 98 | 0.57 | 0.46 | 0.70 | 0.06 | 466 | 5.42 | 4.92 | 5.91 | 0.25 | 688 | 16.73 | 15.48 | 17.99 | 0.64 |
| 2012 | 95 | 0.51 | 0.41 | 0.63 | 0.05 | 469 | 5.33 | 4.84 | 5.82 | 0.25 | 691 | 16.35 | 15.13 | 17.58 | 0.63 |
| 2013 | 93 | 0.52 | 0.42 | 0.63 | 0.05 | 477 | 5.41 | 4.92 | 5.90 | 0.25 | 769 | 17.43 | 16.19 | 18.67 | 0.63 |
| 2014 | 98 | 0.55 | 0.44 | 0.67 | 0.06 | 425 | 4.78 | 4.31 | 5.24 | 0.24 | 731 | 16.26 | 15.07 | 17.45 | 0.61 |
| 2015 | 68 | 0.40 | 0.31 | 0.51 | 0.05 | 419 | 4.70 | 4.24 | 5.16 | 0.23 | 710 | 15.35 | 14.21 | 16.50 | 0.58 |
| 2016 | 82 | 0.47 | 0.37 | 0.58 | 0.05 | 416 | 4.66 | 4.20 | 5.12 | 0.23 | 672 | 13.99 | 12.92 | 15.06 | 0.55 |
| 2017 | 89 | 0.50 | 0.40 | 0.62 | 0.05 | 335 | 3.66 | 3.26 | 4.06 | 0.20 | 730 | 14.66 | 13.58 | 15.73 | 0.55 |
| 2018 | 84 | 0.45 | 0.36 | 0.56 | 0.05 | 348 | 3.82 | 3.41 | 4.23 | 0.21 | 676 | 13.30 | 12.28 | 14.31 | 0.52 |
| 2019 | 75 | 0.40 | 0.31 | 0.50 | 0.05 | 352 | 3.90 | 3.48 | 4.32 | 0.21 | 708 | 13.47 | 12.47 | 14.47 | 0.51 |
| 2020 | 73 | 0.38 | 0.29 | 0.47 | 0.04 | 328 | 3.66 | 3.25 | 4.07 | 0.21 | 651 | 12.05 | 11.12 | 12.99 | 0.48 |
| 2021 | 71 | 0.39 | 0.30 | 0.49 | 0.05 | 303 | 3.32 | 2.94 | 3.71 | 0.20 | 662 | 12.32 | 11.37 | 13.27 | 0.49 |
| 2022 | 56 | 0.29 | 0.22 | 0.38 | 0.04 | 290 | 3.26 | 2.88 | 3.65 | 0.20 | 723 | 12.91 | 11.96 | 13.86 | 0.48 |
| 2023 | 67 | 0.35 | 0.27 | 0.45 | 0.04 | 289 | 3.20 | 2.82 | 3.58 | 0.19 | 649 | 11.27 | 10.39 | 12.14 | 0.45 |

AAMR: age adjusted mortality rates

Supplementary Table S5. AAMR for SSc as the multiple cause of death, by sex, United States, 1999–2023

|  | Total | | | | | Female | | | | | Male | | | | | |
| --- | --- | --- | --- | --- | --- | --- | --- | --- | --- | --- | --- | --- | --- | --- | --- | --- |
| Year | Number | AAMR | 95%CI | | SE | Number | AAMR | 95%CI | | SE | Number | AAMR | 95%CI | | SE |  |
| 1999 | 1941 | 7.06 | 6.75 | 7.38 | 0.16 | 1583 | 10.41 | 9.89 | 10.92 | 0.26 | 358 | 2.89 | 2.59 | 3.20 | 0.16 |  |
| 2000 | 1947 | 7.02 | 6.70 | 7.33 | 0.16 | 1527 | 9.90 | 9.40 | 10.40 | 0.25 | 420 | 3.37 | 3.05 | 3.70 | 0.17 |  |
| 2001 | 1975 | 6.98 | 6.67 | 7.29 | 0.16 | 1603 | 10.25 | 9.74 | 10.75 | 0.26 | 372 | 2.87 | 2.58 | 3.17 | 0.15 |  |
| 2002 | 1923 | 6.69 | 6.39 | 6.99 | 0.15 | 1527 | 9.62 | 9.14 | 10.11 | 0.25 | 396 | 2.99 | 2.70 | 3.29 | 0.15 |  |
| 2003 | 1947 | 6.64 | 6.34 | 6.93 | 0.15 | 1549 | 9.61 | 9.13 | 10.09 | 0.25 | 398 | 2.96 | 2.66 | 3.25 | 0.15 |  |
| 2004 | 1873 | 6.30 | 6.02 | 6.59 | 0.15 | 1523 | 9.34 | 8.86 | 9.81 | 0.24 | 350 | 2.52 | 2.25 | 2.79 | 0.14 |  |
| 2005 | 1962 | 6.47 | 6.18 | 6.76 | 0.15 | 1577 | 9.47 | 9.00 | 9.94 | 0.24 | 385 | 2.72 | 2.45 | 3.00 | 0.14 |  |
| 2006 | 1804 | 5.85 | 5.58 | 6.12 | 0.14 | 1452 | 8.58 | 8.14 | 9.02 | 0.23 | 352 | 2.45 | 2.19 | 2.71 | 0.13 |  |
| 2007 | 1838 | 5.81 | 5.55 | 6.08 | 0.14 | 1475 | 8.54 | 8.10 | 8.98 | 0.22 | 363 | 2.49 | 2.23 | 2.74 | 0.13 |  |
| 2008 | 1801 | 5.62 | 5.36 | 5.88 | 0.13 | 1470 | 8.43 | 8.00 | 8.86 | 0.22 | 331 | 2.24 | 2.00 | 2.49 | 0.13 |  |
| 2009 | 1808 | 5.55 | 5.29 | 5.81 | 0.13 | 1460 | 8.23 | 7.80 | 8.66 | 0.22 | 348 | 2.26 | 2.02 | 2.50 | 0.12 |  |
| 2010 | 1754 | 5.27 | 5.02 | 5.52 | 0.13 | 1428 | 7.85 | 7.44 | 8.26 | 0.21 | 326 | 2.08 | 1.85 | 2.31 | 0.12 |  |
| 2011 | 1734 | 5.10 | 4.85 | 5.34 | 0.12 | 1402 | 7.60 | 7.20 | 8.01 | 0.21 | 332 | 2.10 | 1.87 | 2.33 | 0.12 |  |
| 2012 | 1716 | 4.92 | 4.68 | 5.16 | 0.12 | 1370 | 7.25 | 6.86 | 7.64 | 0.20 | 346 | 2.13 | 1.90 | 2.36 | 0.12 |  |
| 2013 | 1809 | 5.07 | 4.83 | 5.30 | 0.12 | 1470 | 7.56 | 7.16 | 7.95 | 0.20 | 339 | 2.03 | 1.81 | 2.25 | 0.11 |  |
| 2014 | 1727 | 4.78 | 4.55 | 5.01 | 0.12 | 1391 | 7.10 | 6.72 | 7.48 | 0.19 | 336 | 2.00 | 1.78 | 2.22 | 0.11 |  |
| 2015 | 1702 | 4.56 | 4.34 | 4.78 | 0.11 | 1389 | 6.86 | 6.49 | 7.23 | 0.19 | 313 | 1.80 | 1.60 | 2.01 | 0.10 |  |
| 2016 | 1643 | 4.33 | 4.12 | 4.55 | 0.11 | 1341 | 6.56 | 6.20 | 6.92 | 0.18 | 302 | 1.70 | 1.50 | 1.90 | 0.10 |  |
| 2017 | 1682 | 4.37 | 4.15 | 4.58 | 0.11 | 1376 | 6.60 | 6.24 | 6.96 | 0.18 | 306 | 1.69 | 1.50 | 1.89 | 0.10 |  |
| 2018 | 1605 | 4.08 | 3.87 | 4.28 | 0.10 | 1297 | 6.11 | 5.77 | 6.45 | 0.17 | 308 | 1.65 | 1.46 | 1.84 | 0.10 |  |
| 2019 | 1636 | 4.08 | 3.88 | 4.28 | 0.10 | 1302 | 6.01 | 5.67 | 6.34 | 0.17 | 334 | 1.78 | 1.59 | 1.98 | 0.10 |  |
| 2020 | 1669 | 4.09 | 3.89 | 4.29 | 0.10 | 1361 | 6.17 | 5.83 | 6.50 | 0.17 | 308 | 1.66 | 1.47 | 1.85 | 0.10 |  |
| 2021 | 1749 | 4.28 | 4.07 | 4.48 | 0.10 | 1446 | 6.56 | 6.21 | 6.91 | 0.18 | 303 | 1.59 | 1.41 | 1.78 | 0.09 |  |
| 2022 | 1801 | 4.27 | 4.07 | 4.47 | 0.10 | 1433 | 6.27 | 5.93 | 6.60 | 0.17 | 368 | 1.94 | 1.74 | 2.14 | 0.10 |  |
| 2023 | 1621 | 3.79 | 3.60 | 3.98 | 0.10 | 1311 | 5.70 | 5.38 | 6.01 | 0.16 | 310 | 1.53 | 1.36 | 1.71 | 0.09 |  |

AAMR: age adjusted mortality rates
